# Ingestion of diverse protein-rich whole-foods result in similar post exercise whole body and myofibrillar protein synthesis rates compared with a more isolated protein source in young adults

**DOI:** 10.1016/j.ajcnut.2026.101231

**Published:** 2026-02-03

**Authors:** Freyja AD Haigh, Alistair J Monteyne, Doaa R Abdelrahman, Andrew J Murton, Tim JA Finnigan, Hannah E Theobald, Francis B Stephens, Benjamin T Wall

**Affiliations:** 1 Department of Public Health and Sport Sciences, Faculty of Health and Life Sciences, University of Exeter, Exeter, United Kingdom; 2 Department of Surgery, University of Texas Medical Branch, Galveston, TX, United States; 3 Sealy Center of Aging, University of Texas Medical Branch, Galveston, TX, United States; 4 Marlow Foods Ltd, Stokesley, North Yorkshire, United Kingdom

**Keywords:** whole-foods, amino acids, muscle protein synthesis, resistance exercise, protein metabolism

## Abstract

**Background:**

The ingestion of protein-rich whole-foods may stimulate (post exercise) whole body (WB) and myofibrillar protein synthesis (MyoPS) rates to a greater extent than more isolated protein sources.

**Objectives:**

We assessed WB protein turnover and MyoPS rates after a bout of resistance exercise and ingestion of a variety of protein-rich whole-foods (animal and nonanimal) and egg whites (more isolated protein source) in resistance-trained young adults.

**Methods:**

In a randomized parallel group design, 65 healthy individuals received primed, continuous infusions of L-[*ring*-^2^H_5_]phenylalanine and L-[3,3–^2^H_2_]tyrosine and completed a bout of lower-body resistance exercise before ingesting 0.25 g protein per kg body mass (bm) from: egg whites (*n* = 11), egg (*n* = 11), salmon (*n* = 10), pork (*n* = 11) lentils (*n* = 11) or mycoprotein (*n* = 11). Blood and muscle samples were taken pre- and (120 and 300 min) post exercise/food ingestion to determine WB phenylalanine kinetics and MyoPS rates. Calculated WB phenylalanine kinetics and fractional synthesis rates were analyzed using 2-way (group × time) analysis of variance.

**Results:**

WB protein synthesis and breakdown rates increased and decreased, respectively, post exercise/food ingestion in all groups; though a greater positive WB net protein balance was achieved (primarily via greater suppression of breakdown) after egg white ingestion, despite similar insulinemia across all groups [postprandial net balance (0–300 min); egg whites, 256.6 ± 15.4 compared with.; egg, 161.0 ± 6.1; pork, 166.8 ± 7.2; salmon, 195.4 ± 7.1; lentils, 175.8 ± 8.1; mycoprotein, 189.7 ± 8.4 μmol/kg/min (*P* < 0.0001, all)]. MyoPS rates increased after exercise/food ingestion with no (temporal) differences between groups despite divergent plasma amino acid responses [Δ change in fractional synthesis rates (0–300 min); egg whites, 0.050 ± 0.013; egg, 0.051 ± 0.009; pork, 0.008 ± 0.008; salmon, 0.021 ± 0.014; lentils, 0.029 ± 0.012; mycoprotein, 0.041 ± 0.012% per hour (*P* = 0.077)].

**Conclusions:**

The ingestion of a variety of protein-rich whole-foods or a more isolated protein source (egg whites) after lower-body exercise results in comparable MyoPS rates, though a greater WB net protein anabolism was achieved with egg whites.

The trial was registered at clinicaltrials.gov (NCT04794153). Date of Registration: 2021-03-04. https://clinicaltrials.gov/study/NCT04794153?term=NCT04794153&rank=1

Clinical Trial Register No. (clinicaltrials.gov): NCT04794153.

## Introduction

In the absence of sufficient protein ingestion after resistance exercise, net whole body (WB) and muscle protein balance remains negative, limiting the accretion of new proteins and, consequently, adaptive responses over time (e.g., muscle hypertrophy) [1]. Dietary protein ingestion augments post exercise WB and muscle protein synthesis (MPS) rates, suppresses protein breakdown, achieves positive net protein balances, and supports adaptation. The regulation of the post exercise WB and MPS responses to protein ingestion are thought to be contingent on the rise of circulating (essential) amino acids [2], influenced by the quantity [[3], [4], [5]], source [6], and form [7,8] of protein consumed. Current (sports) dietary protein guidelines are largely predicated on evidence derived from mechanistic investigations of isolated protein sources; however, less work has evaluated the anabolic effects of ingesting protein-rich whole-foods [[8], [9], [10], [11], [12]], despite them contributing the majority of daily protein within normal eating patterns, even in trained individuals [13].

Although defining whole food is equivocal, the term typically refers to food sources complete with naturally existing macro and micronutrients contained within a unique food matrix [14]. Although protein has been assumed to be the only fundamentally anabolic nutrient, recent work suggests protein-rich whole-foods may stimulate post exercise MPS rates to a greater extent than protein alone, potentially due to the presence of, or interaction between, more nutrient-dense profiles. The clearest illustration of this was shown by van Vliet et al. [11], who reported that ingestion of whole eggs stimulated greater MPS rates than those for an isonitrogenous dose of egg whites alone. Although this work implies that nonprotein components (or nutrient interactions) within the whole egg matrix may potentiate the anabolic response (coined “the whole-food effect”) [15,16], other analogous studies have been equivocal.

For example, support for a whole-food effect can be found from data reporting a more positive net muscle protein balance following the ingestion of whole milk compared with skimmed milk [17] and (post exercise) mycoprotein ingestion compared with concentrated milk protein [9]. Others, however, have failed to confirm these findings; for instance, whole mycoprotein ingestion compared with protein isolated from mycoprotein [8] and salmon ingestion compared with the same nutrients as an isolated mixture of crystalline amino acids and fish oil [12]. Therefore, a more comprehensive assessment is required to compare a variety of commonly consumed whole-food sources with differing nutritional profiles to properly judge whether a singular whole food source is more anabolic than other sources, and to replicate the clearest example shown thus far (i.e., whole egg ingestion).

The present work assessed the post exercise WB and MPS responses to a variety of protein-rich whole-foods with diverse nutritional profiles (Supplementary Tables 1–3). We selected egg whites as a control (less nutrient-dense and more isolated protein) [11] and compared with whole egg and 4 other groups. Mycoprotein is an established high-quality, sustainable alternative protein [18] but previously not investigated in food form (only as freeze-dried solubilized powder) [8,9,19]. Lentils were included as an additional higher fiber, nonanimal comparator (only previously studied in animals) [20]. Pork is one of the most commonly consumed meats worldwide [21] and was previously reported to provide a robust anabolic stimulus [22], and salmon, based upon its omega-3 fatty acid content, was previously suggested to have anabolic properties [23]. We hypothesized that the ingestion of a variety of protein-rich whole-foods would stimulate post exercise MPS rates to a greater extent than a more isolated protein source and result in a greater WB net protein balance. Given the precise mechanisms of how nonprotein components in whole-foods are incompletely understood, we chose not to directly hypothesize differences between the whole-foods.

## Methods

### Participants

Sixty-five resistance-trained, young, and healthy males (*n* = 41) and females (*n* = 24) volunteered to take part in the present study. Participants were made aware of the study primarily through social media advertisements and posters displayed across the university campus. Participants’ characteristics are presented in Supplementary Table 1 (Supplementary Figure 1 for Participant flow diagram). Participants were considered resistance trained if they were engaged in WB resistance exercise training (≥3 times per week for ≥6 mo) prior to taking part in the study. This population was selected as we wished to homogenize the response, as it has been shown that training status affects the magnitude of the MPS response to protein and exercise [24]. Other exclusion criteria included any metabolic impairment, cardiovascular complications, allergies to mycoprotein/Quorn/edible fungi or environmental molds, or being classified as untrained according to the above criteria. Participants were admitted onto the study after being deemed healthy based on blood pressure (<140/90 mmHg), BMI (18–30 kg/m^2^), and responses to a routine medical health questionnaire. Female participants completed the experimental visit during the first 8 d of their self-reported menstrual cycle (follicular phase) to limit potential differences in protein metabolic responses due to hormonal fluctuations [25]. Experimental procedures, potential risks, and the purpose of the study were explained to the participants before obtaining informed written consent. This study was approved by the Sport and Health Sciences ethics committee of the University of Exeter (210203-B-03) in accordance with the Declaration of Helsinki and is registered at clinicaltrials.gov (NCT04794153). Recruitment and data collection were conducted in the Nutritional Physiology Research Unit at the University of Exeter between June 2021 and February 2024.

Upon acceptance into the study, all participants underwent a pre-testing protocol, completed at least 5 d before the experimental trial. Participants were familiarized with the exercise equipment and exercise protocol (described below in resistance exercise protocol), and measures of body composition (body fat in percentage and lean mass in kilograms) were determined by Air Displacement Plethysmography (BodPod, Life Measurement, Inc).

### Experimental protocol

**FIGURE 1:**
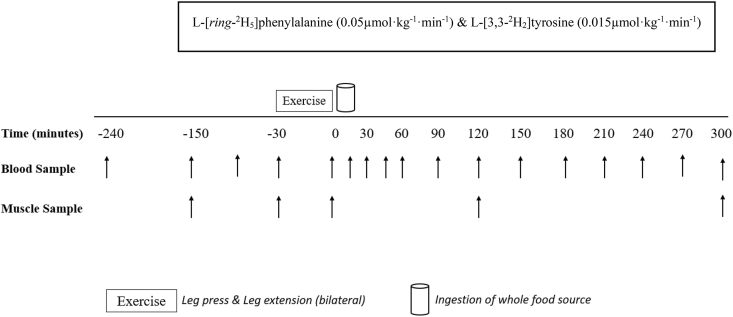
Schematic representation of the experimental visit.

Participants were randomly assigned to 1 of 6 parallel (food) groups. Block randomization was stratified for sex and applied by AJMo. An overview of the experimental protocol can be found in Figure 1. Participants were asked to avoid vigorous physical activity and alcohol in the 48 h preceding the trial. All participants were provided with a standardized meal to consume as their last food intake by 20:00 the day before their experimental visit [1913 kJ (912 kcal), 29% energy from fat, 46% energy from carbohydrate, 25% energy from protein]. On the day of the trial, participants arrived at the laboratory at ∼07:30. A Teflon cannula was inserted into an antecubital vein of one arm for the infusion of stable isotope tracers. Before the infusion was initiated, a baseline venous blood sample was taken from this site to measure background isotopic enrichments. After the baseline blood sample collection (*t* = −240 min), the phenylalanine and tyrosine pools were primed with a single intravenous dose of L-[*ring*-^2^H_5_]phenylalanine (2.12 μmol/kg) and L-[*ring*-3,3–^2^H_2_]tyrosine (0.75 μmol/kg). After the priming dose, a continuous tracer infusion was initiated (*t* = −240 min), at a rate of 0.05 μmol/kg/min for L-[*ring*-^2^H_5_]phenylalanine and 0.015 μmol/kg/min for L-[3,3–^2^H_2_]tyrosine, for the duration of the protocol. Once this infusion was in progress, a second Teflon cannula was inserted retrogradely into a dorsal hand vein of the contralateral arm and placed in a heated hand unit (55°C) to allow for subsequent arterialized venous blood sampling [26]. After 90 min of continuous infusion (*t* = −150 min), arterialized venous blood samples were then taken throughout the remainder of the infusion at the following time points: −150, −90, −30, 0, 15, 30, 45, 60, 90, 120, 150, 180, 240, and 300 min. A baseline muscle biopsy sample was collected 90 mins after the initiation of the continuous infusion (*t* = −150), and a second was collected at *t* = −30 min from the left leg, at least 2 cm distal to the previous incision. These 2 biopsies were used for the calculation of resting postabsorptive myofibrillar protein synthesis (MyoPS) rates. Muscle biopsies were sampled from the mid region of the *m. vastus lateralis* (∼15 cm above the patella) with a modified Bergström suction needle under local anesthesia (2% lidocaine). All biopsy samples were immediately freed from any visible blood, adipose, and connective tissue, frozen in liquid nitrogen (within 30 s), and stored at –80°C until subsequent analysis.

At −30 min, participants were taken to the research gymnasium (adjacent to the laboratory) to execute a bout of lower-body resistance exercise, as described below in resistance exercise protocol.

Immediately after the completion of exercise, a third muscle biopsy was collected from the right leg before consuming 1 of the 6 experimental foods (*t* = 0) (Supplementary Table 4 for food preparation and Supplementary Tables 1–3 for nutritional compositions and Supplementary Material 4 for nutritional analyses methodologies). With the different nutritional compositions of the food sources and the proposed anabolic properties of nonprotein components, a suboptimal protein serving of 0.25 g protein per kg of body mass (BM) (0.25 g/kg/BM/d) of each food source was selected to minimize saturating the anabolic response to feeding, in an attempt to tease out potential differences between sources [27]. Time to complete food consumption was completed within ∼5 minutes*.* Participants then rested in a semi-supine position for 300 min, with further muscle biopsies (sampled from the right leg) collected at 120 and 300 min after food consumption to determine postprandial, post exercise MyoPS rates over an early (i.e., 0-120 min), later (i.e., 120–300 min), and a combined (i.e., 0–300 min) phase.

### Resistance exercise protocol

During the pre-testing visit, each participant performed a 3-repetition max (RM) to estimate bilateral 1RM for leg press and leg extension [28]. Three-RM rather than 1RM was selected as an accurate approach to predict 10RM to minimize safety risks associated with 1RM testing [28]. Strength testing began with a brief warm-up with a low weight on each exercise. Thereafter, participants performed sets of 8, 6, and 5 repetitions at increasing weight before performing a 3RM attempt. Weight was increased for each subsequent attempt, with the final 3RM being recorded as the last weight lifted correctly before a failed attempt (±5 kg from the failed attempt). An estimated value for a 1RM was calculated with 10RM, then estimated as 70% 1RM. This was repeated for the leg extension exercise. Once 3RM testing had finished, participants rested for 5 min and were then asked to complete one set (10 repetitions) on the leg press machine at the calculated 70% 1RM for familiarization and verification purposes.

For the experimental trial, participants completed a brief warm-up of 10 repetitions of unloaded leg press exercise and 10 repetitions at 50% 1RM. Thereafter, participants performed 4 sets of leg press followed by 4 sets of leg extension, each separated by 2 mins rest. The first set was performed at 70% 1RM, and participants were encouraged to work to volitional failure during each set. For subsequent sets, the weight was adjusted if participants were unable to perform a minimum of 8 repetitions (weight decreased) or exceeded 12 repetitions (weight increased). Verbal encouragement was provided throughout.

### Food preparation and palatability

The eggs were organic and locally sourced in Devon, United Kingdom, during the spring months. Pork meat was obtained from the loin and shoulder muscles of an outdoor-reared pig, and both the pork and eggs were supplied by Ben’s Farm Shop, Devon, United Kingdom. Hand-reared salmon was sourced from Wester Ross, Scotland, and organic red lentils were supplied by buywholefoodfoodsonline.co.uk. Mycoprotein was provided by Marlow Foods Ltd, Quorn Foods, Stokesley, United Kingdom. Natural mycoprotein was produced as previously described [18]. For the production of mycoprotein, *Fusarium venenatum* was grown via continuous aerobic flow fermentation, with the addition of carbohydrate and ammonia substrates, under tightly controlled groups (temperature 28–30°C and pH 6.0). The mycelium of the fungus is heat-treated (72–74°C for 30–45 min) to reduce ribonucleic acid concentrations, then further heat-treated at 90°C. The suspended hyphae are then centrifuged, with the resultant solid mass further concentrated by vacuum chilling.

Salmon (filleted) and pork were individually minced using a manual mincer and homogenized. Half of the eggs were lightly whisked together in a large mixing bowl; the rest were separated to retain the whites, which were lightly whisked and homogenized. Mycoprotein was homogenized to a paste during production and stored as small patties. Each food source was separated into weighed aliquots, vacuum-packed, and frozen at −20°C until required. The evening before the experimental visit, a food aliquot was thawed overnight in the refrigerator (5°C). The lentils were prepared and soaked in water the morning of the experimental visit. All food sources were cooked in a water bath at a controlled temperature (details shown in Supplementary Table 3). Once cooked, the food was weighed to provide 0.25 g/kg/BM/d for each individual participant, and 1 g of salt and mixed herbs were added and mixed with a fork before serving.

After the consumption of the food, participants were asked to complete a sensory test evaluation form [29] and a food action rating test [30] relating to the specific meal they consumed (data shown in Supplementary Table 5).

### Blood sample collection and analyses

Ten milliliters of arterialized venous (except the venous baseline sample) blood were collected into a syringe at each time point. Six milliliters of that sample was added to EDTA-containing tubes (BD Vacutainer LH; BD Diagnostics, Nu-Care) and centrifuged for 10 min at 4000 *× g* at 4°C. The plasma supernatant was then removed, aliquoted, and stored at −80°C for later analyses. The remaining 4 mL of blood was added to additional vacutainers (BD vacutainers SST II, BD Diagnostics, Nu-Care) and left upright to clot at room temperature for 30 min and then centrifuged for 10 min at 4000 *× g* at 4°C. The serum supernatant was then removed, aliquoted, and stored at −80°C for future analyses.

Serum insulin concentrations were measured using a commercially available ELISA kit (DRG Insulin ELISA, EIA-2935, DRG International Inc). Plasma L-[*ring*-^2^H_5_]phenylalanine enrichments [mole percentage excess (MPE)] and concentrations of phenylalanine, leucine, valine, isoleucine, lysine, histidine, glutamic acid, methionine, proline, serine, threonine, tyrosine, and alanine were determined in tert-butyldimethylsilyl derivatives by GC-MS with electron impact ionization (Agilent, Santa Clara) as described previously [31]. Briefly, to prepare samples for GC-MS, 10 μL of 2 mM nor-leucine was added as an internal standard to 450 μL plasma and deproteinized on ice with 450 μL of 15% 5-sulfosalicylic acid. Samples were then vortexed and centrifuged at 4000 *× g* for 10 min at 4°C. The supernatant was then loaded onto cation exchange columns. Columns were then filled with ddH_2_O, followed by 6 mL 0.5 M acetic acid and then washed once more with ddH_2_O, with the columns allowed to drain between each step. The amino acids were then eluted with 2 mL of 6 M ammonia hydroxide. The eluate was dried using a Speed-Vac for 8 h at 60°C. Samples were subsequently derivatized and analyzed by GC-MS as described below.

A 7-point standard curve containing norleucine and a mixture of amino acids (alanine, glycine, valine, leucine, isoleucine, proline, methionine, serine, threonine, phenylalanine, aspartic acid, glutamic acid, lysine, histidine, and tyrosine) was routinely performed when analyzing plasma amino acid concentrations. The lower and upper limit of quantification were 0 and 1000 μM, respectively. No samples fell outside of this range. An internal standard (norleucine) to which samples were normalized was included during sample preparation to eliminate the effect of inter- and intra-assay variability. All samples were run in duplicate.

### Muscle tissue analyses

Myofibrillar protein extractions were performed as previously described [32]. Approximately ∼50 mg of muscle tissue was homogenized using a mechanical glass pestle in a glass tube in homogenization buffer (in mM: 50 Tris·HCl pH 7.4, 1 EDTA, 1 EGTA, 10 b-glycerophosphate salt, 50 NaF, and 0.5 activated Na3VO4 [Sigma-Aldrich Company Ltd]) with a complete protease inhibitor cocktail tablet (1 tablet per 50 mL of buffer; Roche).

The homogenate was centrifuged at 2200 × *g* for 10 min at 4°C. The supernatant (sarcoplasmic fraction) was aliquoted and stored at −80°C. The remaining pellet was then washed in 500 μL of homogenization buffer and centrifuged again at 700 × *g* for 10 min at 4°C, and the resultant supernatant was discarded. The remaining protein portion, containing myofibrillar and collagen, was then solubilized in 750 μL of 0.3 M sodium hydroxide and heated at 50°C for 45 mins and centrifuged at 10,000 × *g* for 5 min at 4°C. The supernatant (myofibrillar fraction) was then aliquoted into a new 2 mL Eppendorf and precipitated in 500 μL of 1M perchloric acid. These samples were centrifuged at 700 × *g* for 10 min at 4°C, and the resultant supernatant was discarded. The remaining myofibrillar pellet was washed in 1 mL 70% ethanol and centrifuged at 700 × *g* for 5 min at 4°C before the ethanol was removed. This step was repeated once more before the amino acids were then hydrolyzed by adding 2 mL of 6M hydrochloric acid and heating at 110°C for 24 h. Once hydrolyzed, the amino acids were then dried using a Speed-Vac for 4 h at 80°C. Samples were then reconstituted in 1.5 mL 25% acetic acid and pipetted into the cation exchange column. The Eppendorf was then rinsed again with another 1.5 mL 25% acetic acid. The columns were then eluted with 2 mL of 6M ammonia hydroxide into a 2 mL Eppendorf, and the eluate was dried in Speed-Vac for 8 h at 60°C.

Samples were cleaned by adding 1 mL ddH_2_O and 1 mL 0.1% formic acid in acetonitrile and centrifuged at 10,000 × *g* for 3 min at 4°C. The supernatant was aliquoted into a new Eppendorf and dried in the Speed-Vac for 5 h at 60°C. In order to derivatize the muscle sample, 50 μL N-tert-Butyldimethylsilyl-N-methyltrifluoroacetamide (MTBSTFA) + 1 % tertbutyl-dimethyl chlorosilane and 50 μL acetonitrile were added to the dry samples, vortexed, and heated at 95°C for 45 min [33]. The samples were analyzed by GC-MS (7890 GC coupled with a 5975 MSD; Agilent Technologies) in triplicate using electron impact ionization and selected ion monitoring for measurement of isotope ratios [34]. One microliter of the sample was injected in splitless mode (injector temperature: 280°C). Peaks were resolved using an HP5-MS 30 m × 0.25 mm ID × 0.25 μm capillary column (Agilent). Helium was used as the carrier gas at 1.2 mL/min constant flow rate. The temperature ramp was set from 80–245°C at 11°C/min, then to 280°C at 40°C/min [34]. Selected ion recording groups were used to monitor fragments m/z 237 and 239 for the m+3 and m+5 fragments of phenylalanine-bound protein and m/z 336 and 341 for the m+0 and m+5 fragments of the phenylalanine-free fraction. A single linear standard curve from mixtures of known m+5/m+0 ratios for L-[*ring*-^2^H_5_]phenylalanine was used to determine the enrichments of the protein-bound samples using the m+5/m+3 ratio.

To determine muscle D5-enrichment values, a 7-point standard curve produced from unlabeled and D5-phenylalanine, creating a range of isotope dilutions from 0% to 20% MPE, was routinely performed when analyzing the amino acids of the intracellular and bound muscle protein pools. Isotope enrichment was linear across the examined range. Any samples where D5-phenylalanine was not detected were reextracted and repeated. All samples were analyzed in triplicate, and the mean intra-assay coefficient of variance was 6.3%. All samples were analyzed on an Agilent GC/MS 7890A/5975C inert MSD system.

### Calculations

Intravenous infusion of L-[*ring*-^2^H_5_]phenylalanine and L-[3,3–^2^H_2_]tyrosine, and arterialized venous blood sampling were used to assess WB phenylalanine kinetics under steady (postabsorptive) and nonsteady (postprandial) state conditions [35]. WB total phenylalanine rates of appearance (R_a_), rates of disappearance (R_d_), and hydroxylation rates (the initial step of phenylalanine oxidation) were calculated using modified Steele’s equations as follows [35]:

$$Total\;Ra=\frac{F_{iv}-\left[pV\times C\left(t\right)\times \frac{{dE}_{iv}}{dt}\right]}{E_{iv\;}\left(t\right)}$$

$$Total\;R_{d\;}=R_{a\;}-pV\times \frac{dc}{dt}$$

$$Phe\;Hydroxylation=Tyr\;R_{a}\times \frac{E_{tyr\;}\left(t\right)}{E_{phe}\;\left(t\right)}\times \frac{Phe\;R_{d}}{F_{phe}+Phe\;R_{d}}$$

Where F_iv_ is the intravenous tracer infusion rate (μmol/kg/min), pV (0.125 L/kg) is the distribution volume for phenylalanine [35]. C(t) is the mean plasma phenylalanine concentration between 2 consecutive time points, dE_iv_/dt represents the time-dependent variations of plasma phenylalanine enrichments derived from the intravenous tracer, and E_iv_ (t) is the mean plasma phenylalanine enrichment from the intravenous tracer between 2 consecutive time points. Tyr R_a_ is the total rate of appearance based on L-[3,3–^2^H_2_]tyrosine infusion and plasma enrichment of tyrosine. E_tyr_ (t) and E_phe_ (t) are the mean plasma L-[*ring*-^2^H_4_]tyrosine and L-[*ring*-^2^H_5_]phenylalanine enrichments between 2 consecutive time points, respectively, and F_phe_ is the intravenous infusion rate of L-[*ring*-^2^H_5_]phenylalanine (μmol/kg/min).

The absence of intrinsically labeled protein within the food precluded the differentiation between endogenous and exogenous R_a_. For the calculations of WB phenylalanine breakdown rates, the contribution from exogenous phenylalanine from protein ingestion and tracers infused was subtracted from Total R_a_. For the calculations of WB phenylalanine synthesis rates, phenylalanine hydroxylation was subtracted from the phenylalanine rate of disappearance and applied as follows:

$$Protein\;breakdown\;\left(PB\right)=\left(Total\;Phe\;R_{a}-F_{Phe}\right)-PRO\left(phe\right)$$

$$Protein\;Synthesis\;\left(PS\right)=Phe\;R_{d}-Phe\;hydroxylation$$

Netproteinbalance=ProteinSynthesis−ProteinBreakdown

PRO is the estimated amount of exogenous phenylalanine (g) that appeared in circulation following food consumption, accounting for each participants’ serving of phenylalanine from the specific food (based on 0.25 g/kg/BM/d protein) and splanchnic extraction (29%) of amino acids in young adults [36].

The fractional synthetic rate (FSR) of myofibrillar proteins was calculated using the standard precursor–product method [31], as follows:

$$FSR\;\left(\;\%\;\cdot h^{-1\;}\right)=\lceil \frac{\Delta Ep}{E_{precursor\;\cdot \;t\;}}\rceil \times 100$$

Where ΔE_p_ is the increment in protein-bound L-[*ring*-^2^H_5_]phenylalanine in myofibrillar protein between 2 muscle biopsies, E_precursor_ is the average L-[*ring*-^2^H_5_]phenylalanine enrichment in the plasma precursor pool over time and *t* indicates the time (h) between 2 muscle biopsies. A weighted mean of plasma L-[*ring*-^2^H_5_]phenylalanine enrichments was used to account for expected disturbances in the steady state isotopic enrichment in the precursor pool. This approach was taken to allow the simultaneous assessment of whole-body protein kinetics, which would be precluded by oral provision of the same tracer.

### Statistical analyses

The primary and secondary outcome measures of this study were MyoPS rates and WB protein kinetics, respectively, with tertiary outcomes including circulating amino acid and insulin concentrations. An initial 2-sided power analysis with expected effect sizes estimated from previous research [11] revealed that *n* = 12 in each group were sufficient to detect expected differences in postprandial MyoPS rates between groups [egg whites (control) compared with whole egg/salmon/pork/mycoprotein/red lentils] when using a repeated measures analysis of variance (ANOVA) (*P* > 0.05, power 80%, f = 0.56; G∗power version 3.1.9.2). Factoring in a 10%–15% drop out rate, 84 participants would need to be recruited for the study. After enrolment of 5 participants per group, an interim power assessment was performed to ensure appropriate recruitment and minimize participant burden. A one-way ANOVA on interim MyoPS yielded an R^2^ value of 0.40, which was converted to an estimated effect size of f ≈ 0.82. This effect size was used to reassess the required sample size, indicating that approximately 11 participants per group would provide adequate power to detect expected between-group differences. The study recruited 72 participants with 7 dropouts; therefore, data were presented for *n* = 65. Missing data derived from plasma, serum, or muscle samples were handled using linear interpolation, which occurred at random and was predominantly the result of cannulation failure at individual time points, but only existed in <5% of samples [31,37]. Statistical significance was set at *P* < 0.05. All calculations were performed on GraphPad 10.0. Participants’ characteristics, total work done, and background L-[*ring*-^2^H_5_]phenylalanine enrichments were analyzed using one-way ANOVAs. Differences in serum insulin concentrations, plasma amino acid concentrations, plasma tracer enrichments, L-[*ring*-^2^H_5_]phenylalanine, L-[3,3–^2^H_2_]tyrosine and L-[*ring*-^2^H_4_]tyrosine enrichments, and myofibrillar L-[*ring*-^2^H_5_]phenylalanine enrichments were compared using 2-way [group (egg whites compared with whole egg/pork/salmon/lentils/mycoprotein) × time] repeated measures ANOVA. Separated 2-way ANOVAs were performed on postabsorptive and post exercise postprandial plasma L-[*ring*-^2^H_5_]phenylalanine enrichments.

Myofibrillar FSRs were analyzed using a 2-way (group × time) ANOVA. Total postprandial amino acid availability and insulin were calculated as incremental AUC(iAUC) with baseline set as *t* = 0 and were analyzed using a 1-way ANOVA for total (0–300 min) iAUC and a 2-way (group × time) repeated measures ANOVA for temporal changes (0–120 and 120–300 min). In addition, Total AUC for serum insulin and plasma concentrations of essential, nonessential, branched chain, and total amino acids were analyzed using a one-way ANOVA (0–300 min). All data are expressed as mean ± SEM.

All data were assessed for normality using the Shapiro–Wilk test, and for homogeneity of variance using the Brown–Forsythe test before performing ANOVA. Additionally, influential observations were evaluated for each physiological variable as part of data quality control. The inclusion of multiple outcomes (e.g., several plasma amino acid measures and myofibrillar FSRs) increases the overall risk of type I error. We did not apply additional corrections across these outcomes because they were pre-specified and our primary aim was to detect potential differences between treatments; applying stricter adjustments would substantially increase the risk of type II error. To support interpretation, we also report effect size estimates and 95% confidence for the primary and secondary outcome measures in Supplementary Material 3 to provide a clearer indication of the magnitude of any treatment differences.

## Results

### Participants’ characteristics

No differences in age, height, body mass, BMI, blood pressure, body fat percentage, or lean mass were detected between groups (*P* > 0.05; Table 1). No differences in strength (leg press, leg extension; all *P* > 0.05) or total exercise volume (weight × rep) performed across leg press and leg extension were detected between groups (*P* > 0.05).

**TABLE 1:** Participants’ characteristics

	Egg whites (*n* = 11)	Whole egg (*n* = 11)	Pork (*n* = 11)	Salmon (*n* = 10)	Lentils (*n* = 11)	Mycoprotein (*n* = 11)
Sex (M/F)	7/4	7/4	7/4	6/4	7/4	7/4
Age, y	22 ± 6	24 ± 6	24 ± 6	24 ± 6	26 ± 6	24 ± 7
Height, m	1.80 ± 0.10	1.75 ± 0.09	1.75 ± 0.09	1.74 ± 0.06	1.75 ± 0.09	1.77 ± 0.05
Body mass, kg	77.4 ± 15.8	77.0 ± 9.1	74.7 ± 12.0	75.2 ± 12.0	77.9 ± 10.1	77.4 ± 11.7
BMI, kg/m^2^	23.8 ± 3.0	25.0 ± 2.4	24.0 ± 3.1	24.8 ± 2.5	25.2 ± 1.9	24.7 ± 3.4
Systolic blood pressure, mmHg	129 ± 14	127 ± 13	121 ± 11	125 ± 13	125 ± 10	124 ± 9
Diastolic blood pressure, mmHg	70 ± 8	70 ± 9	63 ± 9	67 ± 9	69 ± 9	67 ± 6
Fat, percentage body mass	19.5 ± 7.3	20.1 ± 8.0	17.4 ± 7.6	21.9 ± 5.5	20.9 ± 7.4	20.9 ± 8.3
Lean mass, kg	60.6 ± 13.2	62.3 ± 12.4	60.2 ± 12.1	56.8 ± 5.1	59.5 ± 9.8	61.0 ± 9.7
Leg press 1RM, kg	281 ± 28	272 ± 23	269 ± 21	301 ± 30	273 ± 21	270 ± 25
Leg extension 1RM, kg	121 ± 11	125 ± 13	128 ± 13	110 ± 9	119 ± 8	108 ± 9
Total exercise volume, kg × rep	11449 ± 1130	10537 ± 913	11150 ± 997	11215 ± 890	11347 ± 857	10362 ± 1142

Baseline participant characteristics are presented as mean ± SD; 1-way ANOVAs were performed to confirm the absence of physiologically meaningful differences between randomized groups (all *P* > 0.05). Leg press, leg extension and total exercise volume are presented as mean ± SEM.

Abbreviation: ANOVA, analysis of variance; 1RM, 1 repetition maximum.

### Serum insulin and plasma amino acid concentrations

**FIGURE 2:**
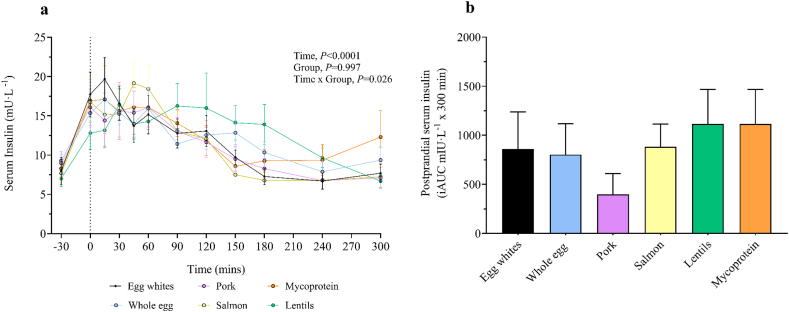
Time course (A) and incremental AUC (iAUC) (B) (calculated as above postabsorptive values) of serum insulin concentrations during a 360-min postabsorptive period (time course graph only showing 30 minutes) and a 300-min postprandial period in young healthy resistance-trained males and females, with iAUCs representing total 300-min postprandial serum concentrations above postabsorptive values. The vertical dashed line indicates the transition from postabsorptive to postprandial groups via the ingestion of 0.25 g per kg of body mass of egg whites (*n* = 11), whole egg (*n* = 11), pork (*n* = 11), salmon (*n* = 10), lentils (*n* = 11), or mycoprotein (*n* = 11), after a single bout of bilaterial lower-body resistance exercise. Time course data were analyzed with a repeated measures 2-way analysis of variance (time × group) with Tukey post hoc tests applied to detect differences at individual time points (time effect; *P* < 0.0001. Group effect; *P* > 0.05. Time × group interaction; *P* = 0.026). iAUC data were analyzed using a 1-way analysis of variance. iAUC, incremental area under the curve. Temporal iAUC of serum insulin concentrations 0–120 min and 120–300 min can be found in Supplementary Figure 2. Values are presented as means, with their SEMs represented by vertical bars.

Complete statistical analyses for serum insulin responses are reported in online Supplementary Table 6, with main effect values shown alongside serum insulin concentrations over the time course of the experiment and visualized in Figure 2. Fasting serum insulin concentrations did not differ between groups (*P* = 0.817). The ingestion of food increased serum insulin concentrations (time effect; *P* < 0.0001), but to differing degrees depending on group (group × time interaction; *P* = 0.026). Total postprandial serum insulin iAUCs did not differ between groups (*P* = 0.624). Temporal iAUC differences between groups were more evident in the late postprandial phase (120–300 min; *P* = 0.005) with the ingestion of lentils eliciting a greater response than with egg whites (*P* = 0.040), pork (*P* = 0.003), and salmon (*P* = 0.026), but with no difference between groups in the early phase (0–120 min; *P* = 0.805), shown in Supplementary Figure 2. In addition, there was no difference between groups for the total AUC for serum insulin concentrations; see Supplementary Figure 3**.**

**FIGURE 3:**
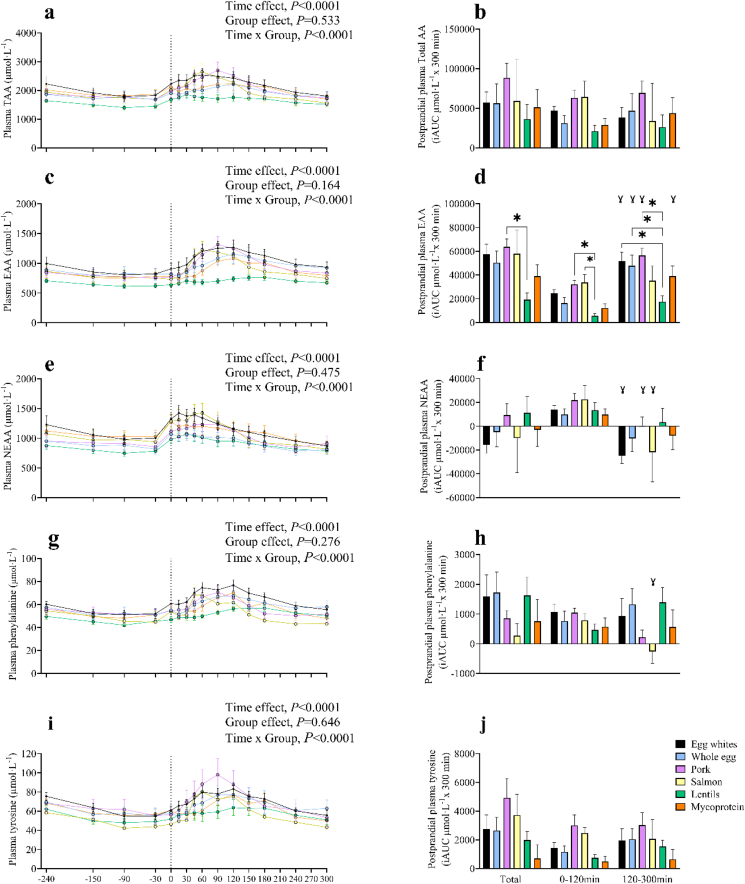
Time course and incremental AUC (iAUC), calculated as above postabsorptive values of plasma total amino acid (A and B), essential amino acid (C and D), nonessential amino acid (E and F), phenylalanine (G and H) and tyrosine (I and J) concentrations during 4-h postabsorptive period (time course graphs only) and a 300-min postprandial period in young healthy resistance-trained males and females. iAUC data represent total 0–300 min, 0–120 min, and 120–300 min postprandial plasma concentrations above postabsorptive values. The vertical dashed line on each graph indicates the transition from postabsorptive to postprandial groups via the ingestion of 0.25 g per kg of body mass of egg whites (*n* = 11), whole egg (*n* = 11), pork (*n* = 11), salmon (*n* = 10), lentils (*n* = 11), or mycoprotein (*n* = 11), where a single bout of bilaterial lower-body exercise was also performed. Time course data were analyzed with a repeated measures 2-way analysis of variance (ANOVA; time × group) with Tukey post hoc tests applied to detect differences at individual time points, which can be found in Supplementary Tables 6 and 7. iAUC data were analyzed using a 1-way ANOVA for total (0–300 min) and a 2-way ANOVA for temporal changes (0–120 min and 120–300 min) with Tukey post hoc tests applied to detect differences at individual time points. TAA, total amino acid; EAA, essential amino acid; NEAA, nonessential amino acid; iAUC, incremental AUC. ∗, represents a significant difference between food groups. ɣ, represents a significant difference between time points (0-120 and 120–300 min). Values are presented as means, with their SEMs represented by vertical bars.

Plasma total amino acid (TAA), essential (EAA), nonessential (NEAA), phenylalanine, and tyrosine concentrations over the time course of the experiment are presented in Figure 3, alongside iAUC for total (0–300 min) and temporal changes (0–120 and 120–300 min). First, postabsorptive plasma TAA, EAA, NEAA, phenylalanine, and tyrosine concentrations remained consistent during the postabsorptive period after the start of the tracer infusion (*P* > 0.05) and did not differ across groups. The ingestion of food increased all values above postabsorptive concentrations (time effects*,* all *P* < 0.0001) and to differing degrees between groups (TAA, EAA, NEAA, phenylalanine, and tyrosine; time × group interactions, all *P* < 0.001). In the first 2 h after food ingestion, iAUC plasma EAA concentrations were lower after the ingestion of lentils compared with pork (*P* = 0.038) and salmon (*P* = 0.036) with differences between groups remaining in the later postprandial phase with the ingestion of lentils resulting in lower EAA concentrations compared with pork (*P* < 0.001), whole egg (*P* = 0.017) and egg whites (*P* = 0.003).

There were no differences between groups in plasma TAA, NEAA, phenylalanine, or tyrosine iAUC during the early, late, or over the total 300 min postprandial periods, displayed in Figure 3. Differences in plasma leucine concentrations did not occur until 60 min after food ingestion (time × group interaction; *P* < 0.0001), with a more rapid increase after the ingestion of egg whites, pork, and whole egg compared with lentils (*P* = 0.038, *P* = 0.013, *P* = 0.046, respectively) and a greater response from pork ingestion compared with mycoprotein (*P* = 0.036). In line, plasma leucine iAUC was significantly greater after the ingestion of egg whites, whole egg, pork, and salmon compared with lentils over the total 300 min postprandial period, as displayed in Supplementary Figure 4. When observing the temporal iAUC changes in plasma leucine concentrations, the ingestion of egg whites, whole egg, and pork resulted in greater plasma leucine concentrations compared with lentils in the late phase (time × group interaction, *P* = 0.041), but not in the early phase (*P* > 0.05), apart from pork, which remained significantly greater over the early (*P* = 0.011) and total 300-min period (*P =* 0.001) yet significantly greater over the total 300-min period (*P* = 0.041). Other individual plasma EAA and NEAA concentrations over the time course of the experiment are presented in Supplementary Figures 4 and 5. All plasma amino acid concentrations changed over time (time effect; *P* < 0.0001). This increase occurred after protein ingestion for all amino acids, with the exception of alanine, which showed a large increase before protein ingestion and consequent to resistance exercise. The results of statistical analyses (*P* values) concerning temporal changes in amino acid kinetics, over time, and for iAUC, are reported in their entirety within online Supplementary Tables 6 and 7, respectively. Total AUC for plasma EAA, NEAA, branched chain amino acid, and TAA concentrations can be found in Supplementary Figure 6.

### Plasma tracers and WB phenylalanine kinetics

**FIGURE 4:**
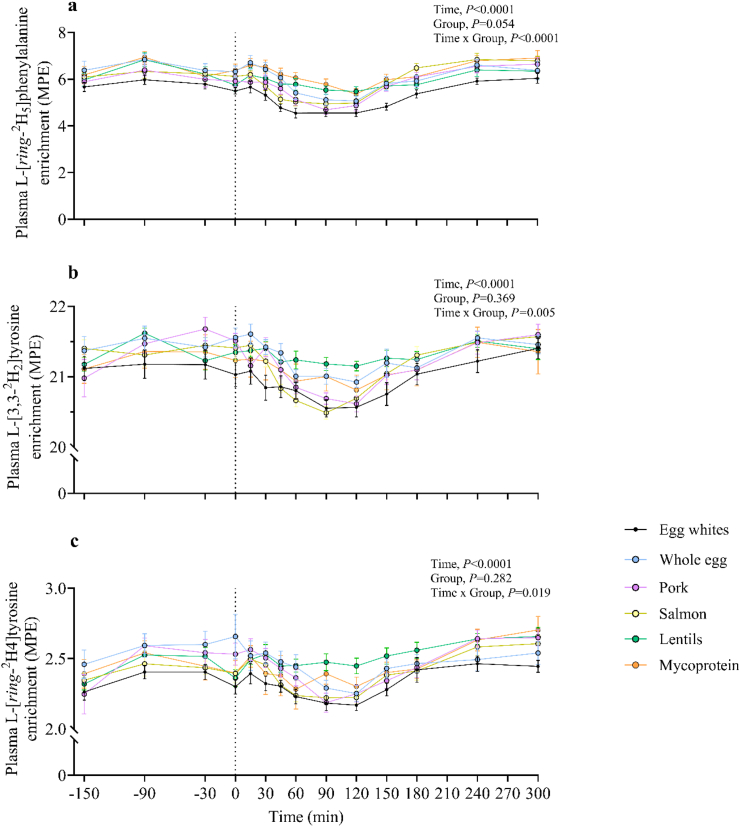
Time course of plasma L-[*ring*-^2^H_5_]phenylalanine (A), L-[3,3–^2^H_2_]tyrosine (B), and L-[*ring*-^2^H_4_]tyrosine (C) enrichments during the experimental trial over a 360-min postabsorptive period (shown as 150 min on graph) and 300 min postprandial period in young healthy resistance-trained males and females. The vertical dashed line indicates the transition from postabsorptive to postprandial groups via the ingestion of 0.25 g per kg of body mass of egg whites (*n* = 11), whole egg (*n* = 11), pork (*n* = 11), salmon (*n* = 10), lentils (*n* = 11), or mycoprotein (*n* = 11), after a single bout of bilaterial lower-body resistance exercise. Time course data were analyzed with a repeated measures 2-way analysis of variance (time × group), with Tukey post hoc tests applied to detect differences at individual time points, which can be found in Supplementary Table 6. Time; all *P* < 0.0001. Group; all *P* > 0.05. Time × group; L-[*ring*-^2^H_5_]phenylalanine *P* < 0.0001, L-[3,3–^2^H_2_]tyrosine *P* = 0.005, L-[*ring*-^2^H_4_]tyrosine *P* = 0.019. Values are presented as means, with their SEMs represented by vertical bars.

The time course of plasma L-[*ring*-^2^H_5_]phenylalanine, L-[3,3–^2^H_2_]tyrosine, and L-[*ring*-^2^H_4_]tyrosine enrichments is illustrated in Figure 4. Postabsorptive plasma L-[*ring*-^2^H_5_]phenylalanine, L-[3,3–^2^H_2_]tyrosine, and L-[*ring*-^2^H_4_]tyrosine did not differ between groups (*P* > 0.05). After protein ingestion, plasma L-[*ring*-^2^H_5_]phenylalanine, L-[3,3–^2^H_2_]tyrosine, and L-[*ring*-^2^H_4_]tyrosine enrichments decreased across all foods (effect of time, *P* < 0.0001, all), but at different rates (time × group interaction effect, *P* < 0.0001, *P* = 0.005, *P* = 0.019, respectively). The results of statistical analyses (*P* values) concerning temporal changes in amino acid enrichments are reported in their entirety within online Supplementary Table 6.

**FIGURE 5:**
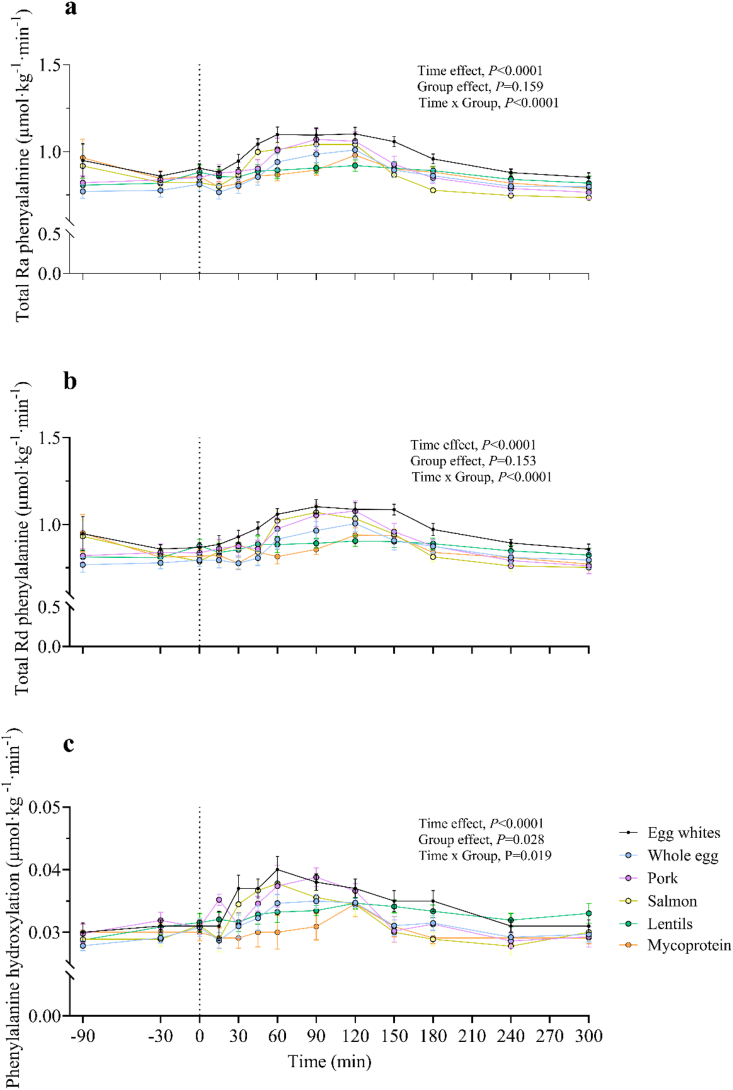
Time course of total phenylalanine rate of appearance (A), total phenylalanine rate of disappearance (B), and phenylalanine hydroxylation (C) during a 360-min postabsorptive (shown as 90 min on graph) and a 300-min postprandial period in young healthy resistance-trained males and females. The vertical dashed line indicates the transition from postabsorptive to postprandial groups via the ingestion of 0.25 g per kg of body mass of egg whites (*n* = 11), whole egg (11), pork (*n* = 11), salmon (*n* = 10), lentils (*n* = 11), or mycoprotein (*n* = 11), after a single bout of bilaterial lower-body resistance exercise. Time course data were analyzed with a repeated measures 2-way analysis of variance (group × time), with Tukey post hoc tests applied to detect differences at individual time points, which can be found in Supplementary Table 6. Time; all *P* < 0.0001. Group; Total R_a_ and Total R_d_, *P* > 0.05; phenylalanine hydroxylation, *P* < 0.05. Time × group; Total R_a_ and Total R_d_, *P* < 0.0001; and phenylalanine hydroxylation, *P* < 0.05. Values are presented as means, with their SEMs represented by vertical bars.

Total phenylalanine rates of appearance (Total R_a_) (representing WB protein breakdown) and total phenylalanine rates of disappearance (Total R_d_) (representing WB protein synthesis + phenylalanine hydroxylation) did not differ between groups in the postabsorptive period (*P* > 0.05), with individual differences between groups appearing 45 min and 60 min after protein ingestion for Total R_a_ and Total R_d_, respectively (time × group, *P* < 0.0001). These individual differences are reported in Supplementary Table 1 and are visualized in Figure 5.

Phenylalanine is oxidized in non-muscle tissues, with the initial step being its hydroxylation to form tyrosine. Intravenous infusions of L-[*ring*-^2^H_5_]phenylalanine and L-[3,3–^2^H_2_]tyrosine were applied, allowing for the measurement of plasma L-[*ring*-^2^H_5_]phenylalanine, L-[3,3–^2^H_2_]tyrosine, and L-[*ring*-^2^H_4_]tyrosine enrichments to assess phenylalanine hydroxylation rates. Phenylalanine hydroxylation rates did not differ between groups in the postabsorptive period (*P* > 0.05); however, there was a significant time (*P* < 0.0001) and group effect (*P* = 0.032), indicative of phenylalanine oxidation rates being increased by food ingestion *per se*, but to a greater extent after 15 min after pork ingestion compared with whole egg (*P* = 0.010) and mycoprotein (*P* = 0.046), with no difference between groups at any other time point (*P* > 0.05).

**FIGURE 6:**
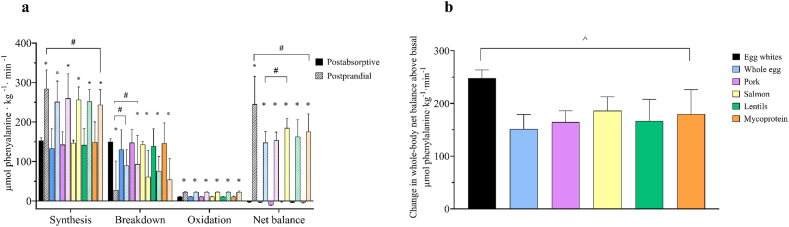
Whole-body phenylalanine kinetics over a 300-min postprandial period (A) and changes in rates of net balance from basal (postabsorptive) in response to the ingestion of 0.25 g per kg of body mass of egg whites (*n* = 11), whole egg (*n* = 11), pork (*n* = 11), salmon (*n* = 10), lentils (*n* = 11), or mycoprotein (*n* = 11), after a single bout of bilaterial lower-body resistance exercise. Data were analyzed using a one-way analysis of variance. ∗Represents a significant difference with ingestion of food; ^#^a significant difference between food groups; and ^ˆ^a significant difference between egg whites and all whole-food groups. Values are presented as means, with their SEMs represented by vertical bars.

Over the total 300 min postprandial period, WB protein synthesis rates increased in all groups from the postabsorptive state (*P* < 0.0001), with rates being greater after egg white ingestion compared with mycoprotein (*P* = 0.046), shown in Figure 6A. WB protein breakdown rates decreased from postabsorptive to post exercise postprandial states in all groups (*P* < 0.0001), apart from whole egg (*P* = 0.298). Protein breakdown rates were suppressed to a greater extent after the ingestion of egg whites compared with pork (*P* = 0.002). Phenylalanine oxidation (hydroxylation) rates increased with food ingestion (*P* < 0.0001), with no difference between groups. WB phenylalanine net balance increased with food ingestion in all groups (*P* < 0.0001), but to a greater extent after the ingestion of egg whites compared with all other groups (*P* < 0.0001, all) and after the ingestion of salmon compared with whole egg (*P* = 0.001), shown in Figure 6B.

### Skeletal muscle tracer analysis

Myofibrillar protein-bound L-[*ring*-^2^H_5_]phenylalanine enrichments did not differ between groups at baseline (egg whites, 0.006 ± 0.002; whole egg, 0.009 ± 0.002; pork, 0.008 ± 0.001; salmon, 0.008 ± 0.002; lentils, 0.009 ± 0.001; mycoprotein, 0.011 ± 0.002, time effect, *P* > 0.05). Post exercise food ingestion increased myofibrillar protein-bound L-[*ring*-^2^H_5_]phenylalanine enrichments to the same extent between groups (time effect *P* < 0.001, time × group *P* = 0.573), at 120 min (egg whites, 0.020 ± 0.003; whole egg, 0.025 ± 0.003; pork, 0.024 ± 0.002; salmon, 0.024 ± 0.002; lentils, 0.024 ± 0.002; mycoprotein, 0.027 ± 0.003) and further increased at 300 min postprandial (egg whites, 0.033 ± 0.002; whole egg, 0.040 ± 0.002; pork, 0.032 ± 0.003; salmon, 0.037 ± 0.003; lentils, 0.036 ± 0.003; mycoprotein, 0.041 ± 0.003).

**FIGURE 7:**
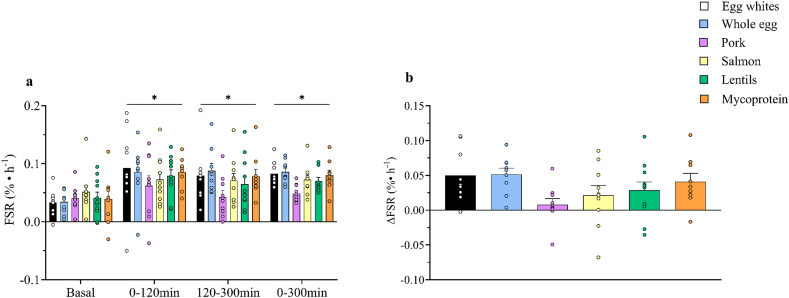
Myofibrillar protein fractional synthesis rates (FSRs) calculated from the plasma L-[*ring*-^2^H_5_]phenylalanine precursor pool in the basal (postabsorptive), temporal postprandial periods (0–120 and 120–300 min) and total (0–300 min) (A) and the delta change in FSRs in response to protein ingestion (B), representing the transition from postabsorptive to postprandial conditions, after a single bout of bilateral lower-body resistance exercise in healthy young males and females. Postprandial state represents a 300 min period following the ingestion of 0.25 g per kg of body mass of egg whites (*n* = 9), whole egg (*n* = 9), pork (*n* = 10), salmon (*n* = 10), lentils (*n* = 11), or mycoprotein (*n* = 9). The sample sizes reported reflect the final number of participants included in the myofibrillar FSR analysis. Missing data imputation was applied only when a single non-sequential data point was missing; participants with missing consecutive biopsy data required for FSR calculation were excluded. Basal (postabsorptive) and temporal postprandial periods were analyzed using a 2-way analysis of variance (ANOVA) (group × time), with Tukey post hoc tests applied to locate individual differences. Time; *P* < 0.0001. Group; *P* = 0.109. Time × group; *P* = 0.834. Postabsorptive FSR and total 0–300 min were analyzed with a 2-way ANOVA. Time; *P* < 0.0001. Group; *P* = 0.368. Time × group; *P* = 0.079. Delta change in FSRs was analyzed using a 1-way ANOVA. There was a trend for a difference in delta change postprandial FSR between groups (*P* = 0.077). ∗Represents a significant difference from basal postabsorptive values to postprandial temporal rates (0–120 and 120–300 min) and total 0–300 min, using separate statistical tests. Values are presented as means, with their SEMs represented by vertical bars.

MyoPS rates increased after post exercise food ingestion during both the early (0–120 min) and late (120–300 min) postprandial phases above postabsorptive values (time effect, *P* < 0.0001); however, there was no difference between groups during the early or late postprandial phase (time × group; *P* = 0.515), shown in Figure 7A. In addition, MyoPS rates over the total postprandial period (0–300 min) were greater than postabsorptive rates (time effect; *P* < 0.0001); however, there was no difference between groups over time (time × group; *P* = 0.079). The delta postabsorptive to postprandial rise in MyoPS rates did not differ between groups over the entire 300 min postprandial window [egg whites, 0.050 ± 0.013 %/h; whole egg, 0.051 ± 0.009 %/h; pork, 0.008 ± 0.008 %/h; salmon, 0.021 ± 0.014 %/h; lentils, 0.029 ± 0.012 %/h; mycoprotein, 0.041 ± 0.012 %/h (*P* = 0.077)], shown in Figure 7B.

## Discussion

Inconsistencies have been reported in the few recent studies that have assessed the post exercise anabolic capacity of protein-rich whole-foods compared with more isolated proteins [8,9,11,12,38]. We sought to provide a comprehensive study of post exercise WB net protein anabolism and MyoPS rates after the ingestion of a diverse range of animal and nonanimal protein-rich whole-foods. We hypothesized that ingestion of these foods (egg, pork, salmon, lentils, and mycoprotein) would stimulate greater rates of post exercise WB protein anabolism and/or MyoPS rates over a 5-h period than that of a more isolated source (egg whites). Expectantly, we saw disparate postprandial plasma amino acid responses across the food sources and some alterations in postprandial amino acid metabolic fluxes (discussed in more detail below). However, contrary to our hypothesis, we observed broadly similar anabolic responses at the WB and myofibrillar concentration to post exercise food ingestion across groups; though, paradoxically, we report evidence of egg white ingestion facilitating a more positive post exercise WB net protein balance compared with the whole-foods.

Nutritional strategies to augment post exercise (muscle) reconditioning have largely focused on the ingestion of isolated dietary proteins, with the goal of maximizing the speed and magnitude of aminoacidemia, assumed to positively regulate WB and MyoPS responses [39,40]. However, adults only obtain small (∼8%) amounts of daily protein intake from isolated sources, with the remainder coming from protein-rich whole-foods, far less studied with respect to the regulation of *in vivo* protein metabolism [13]. Consistent with prior data from ourselves (e.g., [19,41,42] and others [10,38,43], in this study, we observed divergent hyperaminoacidemia between whole-food sources, largely attributable to their varying amino acid contents. To illustrate, the EAA content per serving of pork, ∼8.33 g > mycoprotein, ∼7.52 g > salmon, ∼7.46 g > whole egg, ∼7.23 g > lentils, ∼6.07 g > egg whites, ∼4.82 g and the postprandial circulating amino acid responses with respect to their peak concentration and overall availability largely tracked one another (Figure 3; Supplementary Figures 4 and 5; with the same being true for most individual EAAs). However, clearly digestibility factors were also relevant; for example, TAA varied across sources despite being provided in isonitrogenous amounts, with lentils showing the lowest overall availability of TAA and all individual amino acids. This is likely explained by plant-origin foods typically providing more additional macro-nutrient load (and thus more energy), fiber, tannins, and/or phytochemicals (some specific to lentils [44] Supplementary Table 3), which have all been reported to reduce speed and/or total protein digestibility [45].

Despite this, we observed post exercise MyoPS responses to the ingestion of all foods of a magnitude (∼40 %) in line with most other studies of whole-food ingestion [8,11,12,38], and not potentiated nor attenuated relative to a less nutritionally dense more isolated protein source. This disassociation between circulating amino acid [specifically leucine (Supplementary Figure 2)] availability and the magnitude of (post exercise) MyoPS rates aligns with 2 recent systematic reviews [46,47], and likely speaks to the more complex regulation of MyoPS by whole-foods, reviewed previously [16,48]. The starkest illustration of this in the present data is considering the greatest postprandial amino acid availability after pork ingestion compared with the least after lentil ingestion, yet post exercise MyoPS rates were similar. In fact, though statistically not discernible within the current study design, visually (and demonstrable with individual *t*-tests) pork ingestion resulted in the lowest delta change in MyoPS rates.

At the WB concentration, (FIGURE 5, FIGURE 6) resistance exercise and food ingestion increased protein synthesis (by ∼44 %) and phenylalanine hydroxylation rates (i.e., protein oxidation; by ∼51%; Figure 6), and suppressed protein breakdown rates (by ∼52 %); likely as a consequence of sufficient postprandial rises in circulating insulin [49,50], together achieving a more positive net protein balance. Although these WB responses were broadly similar across the food sources, an interesting exception was that protein breakdown rates decreased to the greatest extent after egg white ingestion, conceivably due to greater insulinemia in the early postprandial phase compared with the whole-foods [51]. Alternatively, the similar insulin responses over the entire postprandial period across the more isolated protein and whole-food sources may indicate differential regulation; for example, the speed of amino acid (leucine) arrival into the circulation may play an additional regulatory role in non-muscle tissues [52]. It was also true that egg whites provoked a modestly greater WB protein synthetic response, which, when paired with the potent inhibition of protein breakdown, achieved the greatest post exercise net protein anabolism. It may be speculated, therefore, the more rapid postprandial amino acid (and insulin) responses to a more isolated protein may promote greater rates of anabolism and/or reconditioning in non-muscle tissues. A final observation from our WB data was that pork ingestion elicited greater postprandial amino acid oxidation rates over the first 2 h. This is potentially attributable to prioritizing amino acids for whole-body energy requirements, with participants having spent considerable time in the postabsorptive state (though not easily reconcilable with this not being a universal observation across sources), again indicating amino acid availability may have more complex regulatory roles beyond muscle protein turnover [53].

It is difficult to reconcile why we did not reproduce the findings of van Vliet et al. [11] within aspects of our similar study design (i.e., egg compared with egg whites). Indeed, even with a forced *t*-test comparing only the egg conditions, we still observed no differences in MyoPS rates. Subtle differences in our study design could be explanatory. Candidates include differing tracer approaches (e.g., lighter molecular mass and thus potentially less sensitive and/or not calculated from intrinsically labeled sources), inclusion of female participants increasing heterogeneity, or parallel design precluding a within-subject comparison. Additional experimental considerations of the present work are also worthy of mention. We have previously observed no (post exercise) anabolic advantage of mycoprotein ingested within its natural food matrix compared with its (more) isolated form [8]. We postulated that the larger protein dose (25 g) provided in that study may have saturated the response, impeding our ability to detect subtle differences. We remedied this limitation here by providing what we considered to be a suboptimal dose (∼17 and ∼21 g in females and males, respectively), especially when combined with the systemic amino acid demand of a post exercise period where large amounts of muscle mass had been engaged. We also collected additional biopsies to get a higher resolution of temporal MyoPS responses. However, as discussed, we revealed no differences at the muscle or WB concentration. Our work does not rule out complex and diverse regulation of differing whole-foods within alternative study designs and/or with the selection of different foods or cooking methods/textures [54,55]. Nevertheless, we would offer that our data have important implications for refining dietary protein guidelines to include the discussion of whole-foods for protein anabolism.

Finally, although our FSR data, presented as individual data points, show variability with occasional physiologically implausible values, these represent the natural variability of this in vivo measurement (discussed previously [56]). It must be acknowledged that the final sample sizes were also reduced in some groups after the exclusion of participants with incomplete sequential isotopic enrichment data, which also affected statistical power. We selected the plasma precursor pool, given that it remains the most widely adopted surrogate and comparable method for assessing both MyoPS rates and WB protein kinetics, and thus capturing the inherent biological and methodological variations. It is worth noting that, despite large differences in postprandial plasma amino acid concentrations, intramuscular free amino acid concentrations across food sources may have been similar, which could explain the lack of observed differences in MyoPS rates. Quantifying intramuscular free amino acid concentrations (particularly leucine) represents an important avenue for future work [48,57].

To conclude, the present investigation demonstrated that the ingestion of a modest dose of a diverse range of isonitrogenous protein-rich whole-foods supported comparable post exercise MyoPS rates, which were also not different from a (more) isolated protein source, egg whites, over either an acute (120 min) or more prolonged (300 min) postprandial period. We therefore do not confirm the proposition of the existence of a beneficial anabolic effect of consuming protein as a whole food, and actually observed evidence that WB net anabolism was greater post exercise after egg white ingestion compared with whole-foods. Within the confines of this study design, we show that to support post exercise reconditioning, a more isolated protein or protein-rich whole food, from diverse origins, are all adequate options, with no obvious detriments within those investigated herein.

## Author contributions

The authors’ contributions were as follows – FADH, AJMo, FBS and BTW: designed the research; FADH and AJMo: conducted the research; FADH, DRA and AJMu: performed the biological analysis; FADH and BTW analyzed the data and wrote the manuscript. BTW: has primary responsibility for the final content; and all authors: read and approved the final content.

## Data availability

Data described in the manuscript, code book, and analytic code will be made available upon request pending approval by the corresponding author.

## Funding

This project was sponsored by Marlow Foods Ltd (BTW; grant holder). The private partners have contributed to the project through regular discussion of the work. FADH was supported by a studentship grant in collaboration with Marlow Foods Ltd.

## Conflict of interest

TJAF and HET, at the time of the study design was an employee of Marlow Foods. BTW, FBS and AJMo are employees of the University of Exeter. All other authors report no conflicts of interest.
