# Supplementary material for: Ingestion of diverse protein-rich whole-foods result in similar post exercise whole body and myofibrillar protein synthesis rates compared with a more isolated protein source in young adults
Source: Am J Clin Nutr. 2026 Feb 3;123(4):101231. doi: 10.1016/j.ajcnut.2026.101231 (PMC13084606; doi:10.1016/j.ajcnut.2026.101231)
Supplement: Multimedia component 1 [file mmc1.docx]

**Ingestion of diverse protein-rich whole-foods result in similar post-exercise whole-body and myofibrillar protein synthesis rates compared with a more isolated protein source in young adults**

Freyja AD Haigh

Online Supplemental Data

| Macronutrient | Egg whites | Whole egg | Pork | Salmon | Lentils | Mycoprotein |
| --- | --- | --- | --- | --- | --- | --- |
| Energy, kJ | 239.0 | 580.0 | 600.0 | 848.0 | 382.0 | 352.0 |
| Energy, Kcal | 56.0 | 139.0 | 143.0 | 204.0 | 91.0 | 77.0 |
| *Per 80kg individual* | *106.0* | *192.0* | *153.0* | *201.0* | *202.0* | *133.0* |
| Protein, g | 10.6 | 14.5 | 18.7 | 20.3 | 9.0 | 11.6 |
| *Per 80kg individual* | *20.0* | *20.0* | *20.0* | *20.0* | *20.0* | *20.0* |
| Carbohydrate, g | 2.9 | <0.1 | 1.0 | 0.6 | 7.6 | 1.9 |
| *Per 80kg individual* | *4.0* | *<0.1* | *1.1* | *0.6* | *16.9* | *3.3* |
| Total sugars, g | 0.2 | <0.1 | 0.4 | <0.1 | 0.4 | 0.2 |
| *Per 80kg individual* | 0.2 | <0.1 | 0.4 | <0.1 | 0.9 | 0.3 |
| Fat, g | 0.2 | 9.1 | 7.0 | 13.3 | 0.2 | 1.5 |
| *Per 80kg individual* | *0.2* | *12.6* | *7.5* | *13.1* | *0.4* | *2.6* |
| Saturates, g | <0.1 | 2.8 | 2.6 | 2.3 | <0.1 | 0.3 |
| Mono-unsaturated fats, g | <0.1 | 4.1 | 3.1 | 5.2 | <0.1 | 0.3 |
| Polyunsaturated fats, g | <0.01 | 1.8 | 0.9 | 5.2 | 0.1 | 0.9 |
| Starch, g | 2.7 | <0.1 | 0.6 | 0.6 | 7.1 | 1.6 |
| Dietary Fibre, g | <0.1 | 1.7 | 0.6 | <0.1 | 11.5 | 4.9 |
| *Per 80kg individual* | *<0.1* | *2.3* | *0.6* | *<0.1* | *25.6* | *8.4* |
| Soluble Dietary Fibre, g | 0.0 | 1.2 | 0.0 | 0.0 | 0.0 | 0.0 |
| Insoluble Dietary Fibre, g | 0.0 | 0.5 | 0.6 | 0.0 | 11.5 | 4.9 |

**Supplementary Table 1.** The nutritional composition of the foods per 100g and for the *average 80kg participant* (egg whites, whole egg, pork, salmon, lentils, mycoprotein)

Premier Analytical Services were responsible for determining macronutrient content within each food source. For each method of this analyses please see Supplementary Material 4.

**Supplementary Table 2.** The amino acid composition of the foods per 100g / *for 80kg individual* (egg whites, whole egg, pork, salmon, lentils, mycoprotein).

| Amino acid (g) | Egg whites | Whole egg | Pork | Salmon | Lentils | Mycoprotein |
| --- | --- | --- | --- | --- | --- | --- |
| Tryptophan | 0.20 | 0.21 | 0.23 | 0.25 | 0.07 | 0.17 |
| Histidine | 0.20 | 0.21 | 0.23 | 0.25 | 0.07 | 0.17 |
| Threonine | 0.52 | 0.63 | 0.92 | 0.91 | 0.29 | 0.58 |
| Valine | 0.63 | 0.69 | 0.78 | 0.81 | 0.31 | 0.53 |
| Methionine | 0.40 | 0.41 | 0.48 | 0.55 | 0.06 | 0.20 |
| Lysine | 0.80 | 0.89 | 1.75 | 1.72 | 0.54 | 0.86 |
| Iso-leucine | 0.47 | 0.53 | 0.67 | 0.64 | 0.24 | 0.42 |
| Leucine | 0.90 | 1.02 | 1.50 | 1.39 | 0.54 | 0.79 |
| Phenylalanine | 0.65 | 0.57 | 0.81 | 0.77 | 0.38 | 0.53 |
| **Total EAAs** | **4.77** | **5.16** | **7.37** | **7.29** | **2.50** | **4.25** |
| *Per 80kg individual* | *4.82* | *7.23* | *8.33* | *7.46* | *6.07* | *7.52* |
| Aspartic acid | 1.23 | 1.35 | 2.00 | 2.03 | 0.95 | 1.14 |
| Serine | 0.83 | 0.99 | 0.85 | 0.87 | 0.44 | 0.62 |
| Glutamic acid | 1.51 | 1.66 | 2.92 | 2.60 | 1.31 | 1.31 |
| Alanine | 0.67 | 0.71 | 1.15 | 1.17 | 0.33 | 0.69 |
| Arginine | 0.63 | 0.79 | 1.23 | 1.14 | 0.64 | 0.74 |
| Proline | 0.43 | 0.52 | 0.91 | 0.74 | 0.36 | 0.53 |
| Cystine | 0.25 | 0.27 | 0.18 | 0.17 | 0.07 | 0.09 |
| Tyrosine | 0.46 | 0.51 | 0.69 | 0.66 | 0.23 | 0.46 |
| Glycine | 0.38 | 0.41 | 0.94 | 0.87 | 0.30 | 0.50 |
| **Total NEAAs** | **6.39** | **7.21** | **10.87** | **10.25** | **4.63** | **6.08** |
| *Per 80kg individual* | *6.39* | *9.94* | *11.63* | *10.1* | *10.29* | *10.48* |
| **Total BCAAs** | **2.00** | **2.24** | **2.95** | **2.84** | **1.09** | **1.74** |
| *Per 80kg individual* | *2.00* | *3.09* | *3.16* | *2.80* | *2.42* | *3.00* |

Premier Analytical Services were responsible for determining amino acid content within each food source. For each method of this analyses please see Supplementary Material 4.

**Supplementary Table 3.** The micronutrient composition of the foods per 100g (egg whites, whole egg, pork, salmon, lentils, mycoprotein)

| Micronutrients | | Egg whites | Whole egg | Pork | Salmon | Lentils | Mycoprotein |  |
| --- | --- | --- | --- | --- | --- | --- | --- | --- |
|  |  |  |  |  |  |  |  |  |
| Omega-3, g | | <0.01 | 0.1 | 0.1 | 3.0 | 0.0 | 0.1 |  |
| Omega-6, g | | <0.01 | 1.6 | 0.8 | 2.2 | 0.1 | 0.8 |  |
| Sodium ICP, g | | 0.2 | 0.2 | 0.1 | 0.0 | <0.01 | <0.01 |  |
| Calcium, mg | | 7.3 | 44.3 | 7.7 | 4.0 | 8.4 | 29.4 |  |
| Iron, mg | | <0.03 | 1.5 | 1.0 | 0.3 | 1.8 | 0.4 |  |
| Magnesium, mg | | 13.6 | 12.4 | 22.9 | 27.8 | 22.5 | 36.6 |  |
| Phosphorus, mg | | 13 | 199 | 208 | 270 | 112 | 213 |  |
| Potassium, mg | | 127.6 | 119.9 | 358.6 | 410.9 | 277.0 | 56.7 |  |
| Manganese, mg | | <0.03 | <0.03 | <0.03 | <0.03 | 0.3 | 4.8 |  |
| Copper, mg | | <0.03 | <0.03 | 0.0 | <0.03 | 0.2 | 0.4 |  |
| Zinc, mg | | <0.02 | 1.2 | 3.1 | 0.4 | 1.1 | 8.4 |  |
| Niacin (Tot VitB3), mg | | <0.1 | <0.1 | 6.5 | 11.3 | 1.0 | 0.7 |  |
| Nicotinamide, mg | | <0.1 | <0.1 | 6.1 | 10.5 | 0.8 | 0.3 |  |
| Vitamin B2, mg | | 0.1 | 0.3 | 0.2 | 0.1 | 0.0 | 0.2 |  |
| Vitamin B1, mg | | <0.05 | 0.1 | 0.7 | 0.1 | 0.2 | <0.05 |  |
| Vitamin A, µg | | <10 | 82 | <10 | <10 | <10 | <10 |  |
| Retinol, µg | | <10 | 78 | <10 | <10 | <10 | <10 |  |
| β carotene, µg | | <10 | 20 | <10 | <10 | <10 | 11 |  |
| Vitamin E, mg | | <0.1 | 2.2 | <0.1 | 3.3 | 0.2 | 0.4 |  |
| Vitamin D3 / D2*, µg | | <0.3 | <0.3 | <0.3 | 4.3 | <0.3* | <0.3* |  |
| Vitamin C, mg | | <0.1 | <0.1 | 0.6 | 0.2 | <0.1 | <0.1 |  |
| Vitamin B6, mg | | <0.05 | 0.1 | 0.3 | 0.3 | <0.05 | 0.1 |  |
| Vitamin B12, µg | | <0.1 | 2.3 | 0.7 | 2.7 | <0.1 | <0.1 |  |
| Cholesterol, mg  Premier Analytical Services were responsible for determining micronutrient content within each food source. For each method of this analyses please see Supplementary Material 4. | | <0.5 | <0.5 | 60.4 | 54.4 | <0.5 | <0.5 |  |
|  |  |  |  |  |  |  |  |  |

**Supplementary Table 4.** Cooking preparation of food sources.

| Food source | Egg whites | Whole egg | Pork | Salmon | Lentils | Mycoprotein |
| --- | --- | --- | --- | --- | --- | --- |
| Cooking time  (mins) | 35 | 35 | 45 | 45 | 60 | 45 |
| Cooking temperature (°C) | 80 | 80 | 70 | 70 | 90 | 60 |
| Weight of food served (grams) | 182.3 ± 35.6 | 132.8 ± 14.9 | 100.3 ± 15.2 | 93.1 ± 13.1 | 216.1 ± 27.0 | 166.3 ± 25.1 |

**Supplementary Table 5.** Food action rating and Sensory Test following consumption of food (egg whites, whole egg, pork, salmon, lentils, mycoprotein).

| Food source | Food action rating test (1-7) | Sensory Test | | | | |
| --- | --- | --- | --- | --- | --- | --- |
|  |  | Appearance | Taste | Texture | Aroma | Overall Acceptability |
| Egg whites | 4 ± 1 | 6 ± 1 | 6 ± 2 | 5 ± 2 | 6 ± 1 | 6 ± 1 |
| Whole egg | 4 ± 2 | 5 ± 2 | 6 ± 2 | 6 ± 2 | 7 ± 2 | 6 ± 2 |
| Pork | 3 ± 1 | 5 ± 2 | 7 ± 1 | 6 ± 2 | 7 ± 1 | 7 ± 2 |
| Salmon | 2 ± 1 | 7 ± 1 | 8 ± 1 | 7 ± 1 | 7 ± 2 | 8 ± 1 |
| Lentils | 4 ± 1 | 6 ± 1 | 7 ± 1 | 6 ± 1 | 7 ± 1 | 7 ± 1 |
| Mycoprotein | 5 ± 2 | 4 ± 2 | 4 ± 2 | 4 ± 2 | 5 ± 1 | 5 ± 2 |

Food action rating test scale 1-7; 1. I would eat this every opportunity, 2. I would eat this very often, 3. I like this and would eat it now and then, 4. I would eat this if available but would not go out of my way, 5. I don’t like this but would eat it on occasion, 6. I would hardly ever eat this, 7. I would eat this only if forced to. Sensory test score 1-9. 1. Dislike extremely, 2. Dislike very much, 3. Dislike moderately, 4. Dislike slightly, 5. Neither like or dislike, 6. Like slightly, 7. Like slightly, 8. Like very much, 9. Like extremely.

**Supplementary Figure 1.**


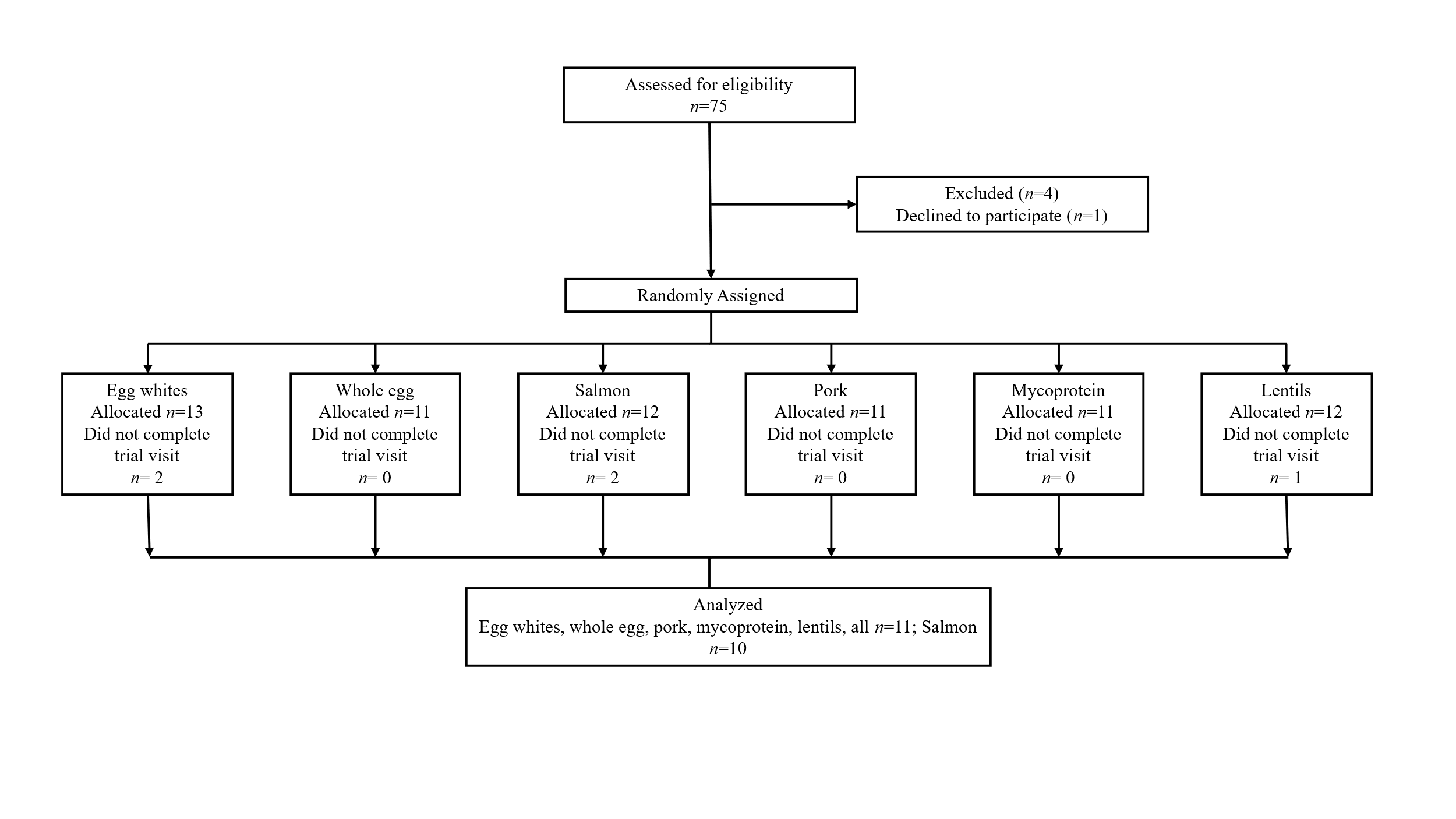


**Supplementary Figure 2.**


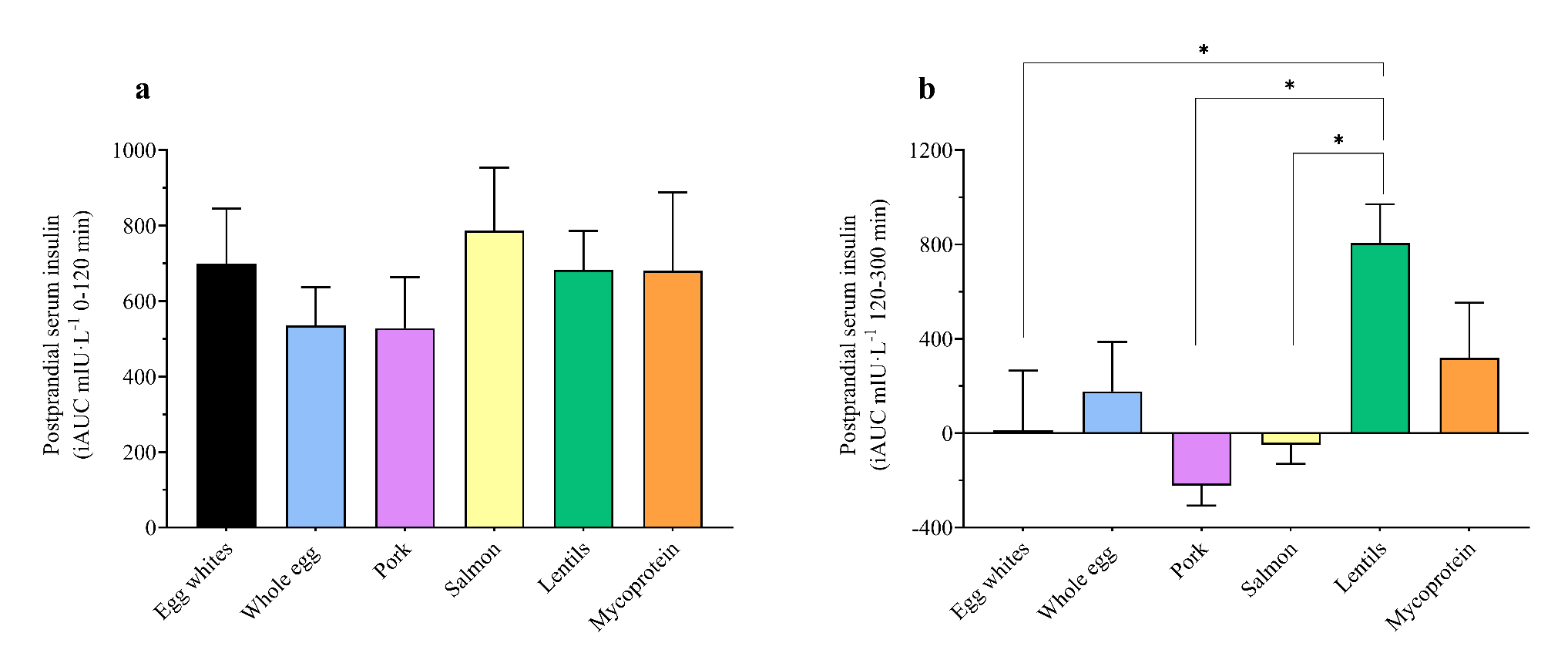


**Supplementary Figure 3.**


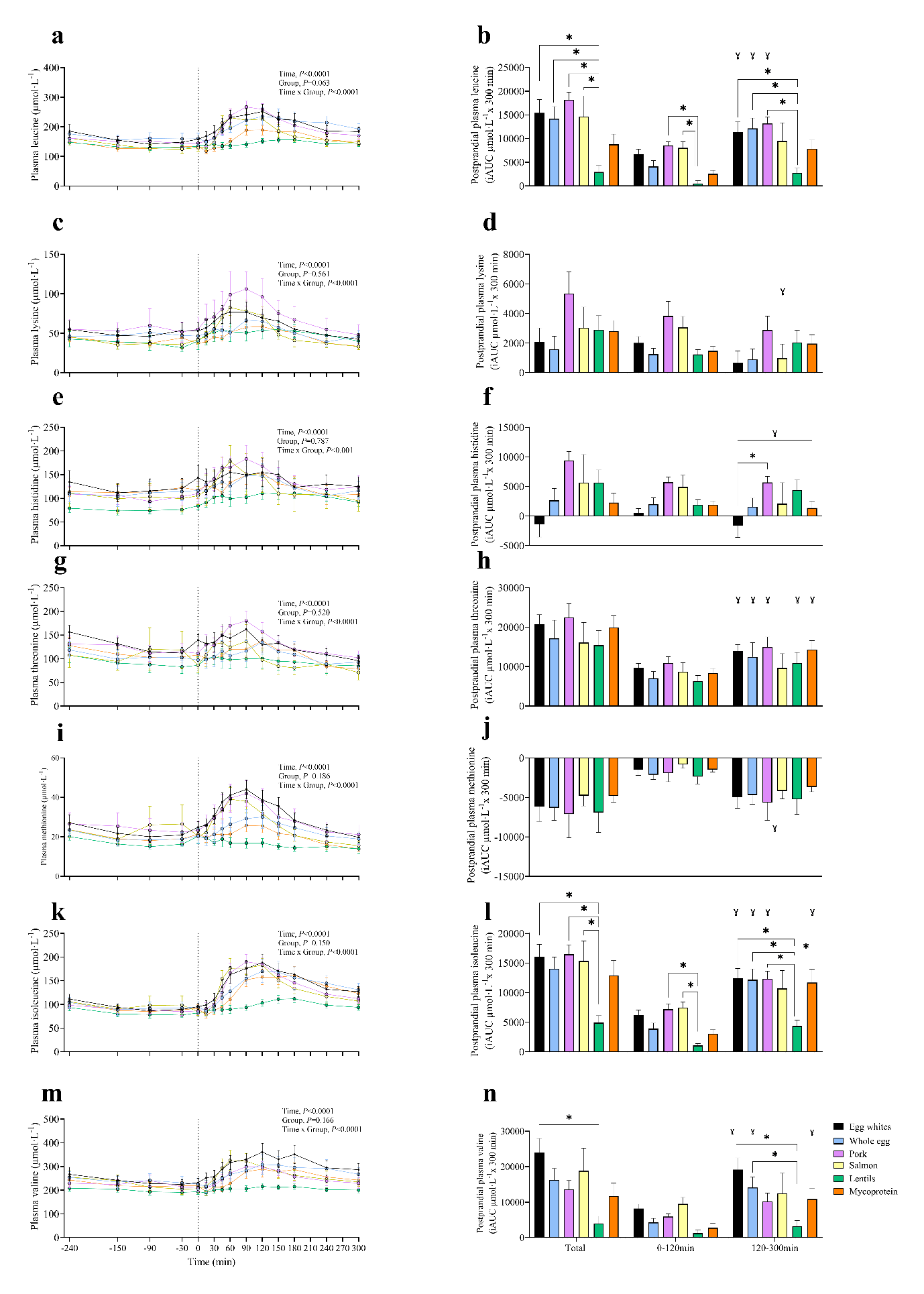


**Supplementary Figure 4.**


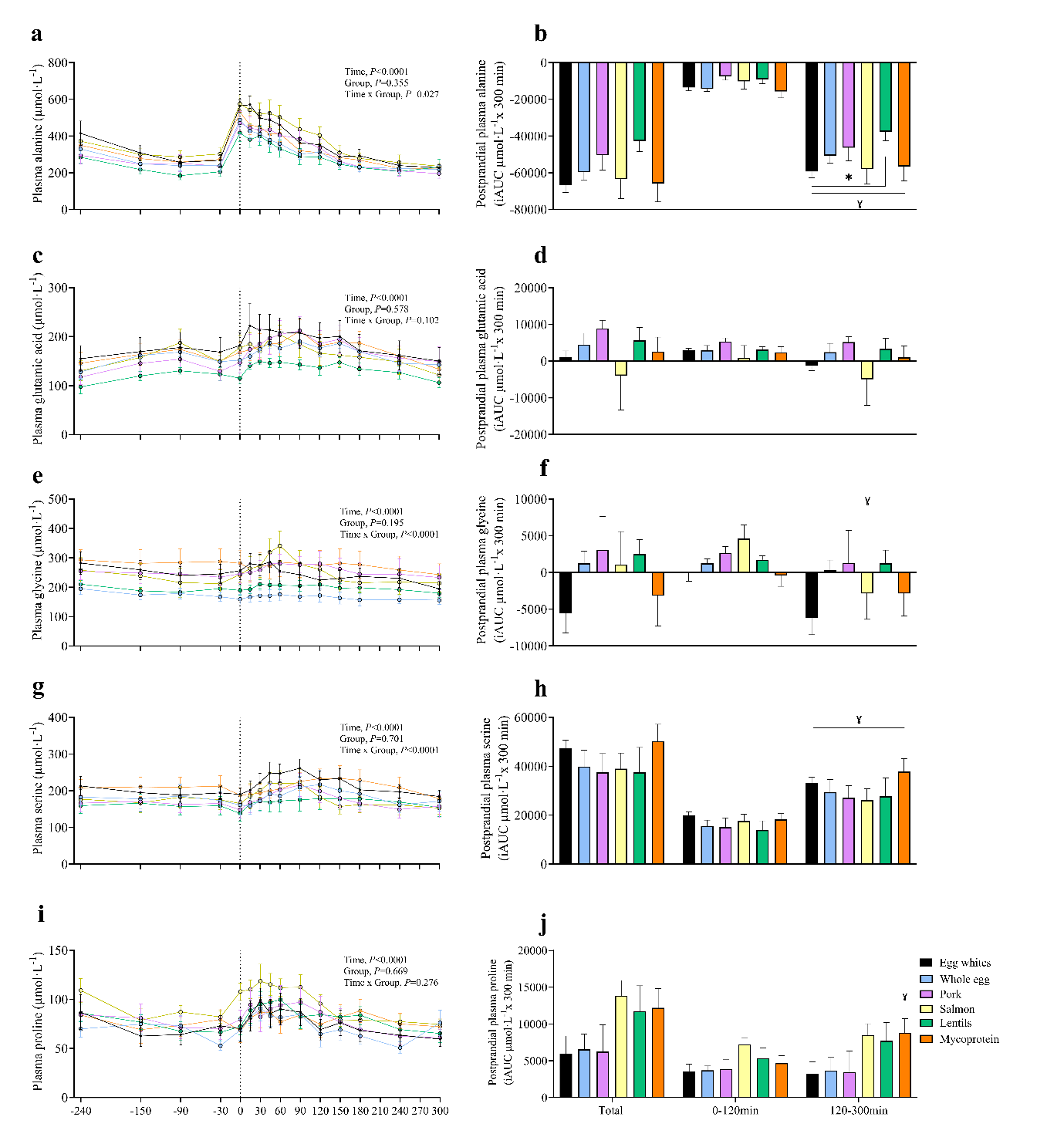


**Supplementary Figure 5.**

**
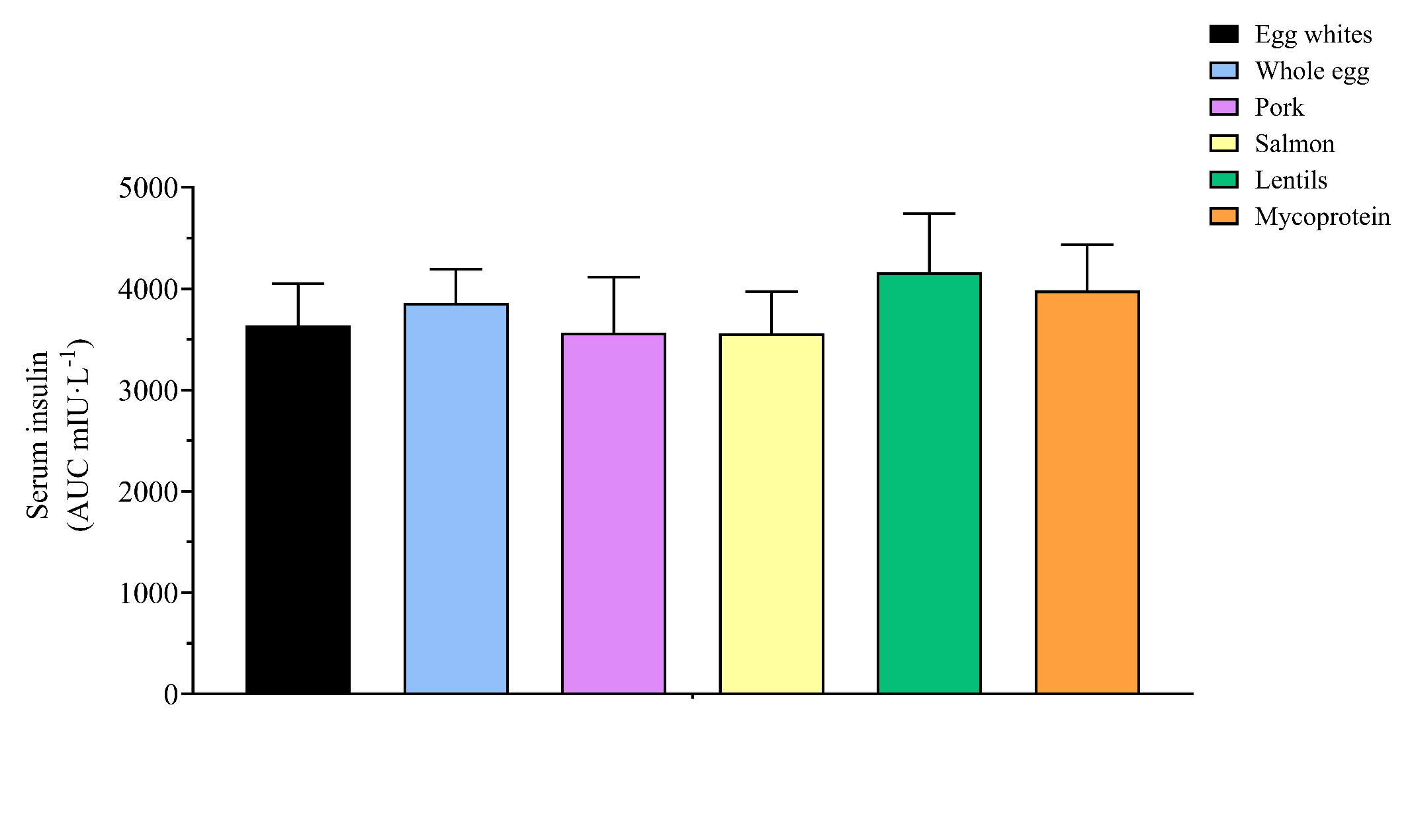
**

**Supplementary Figure 6.**

**
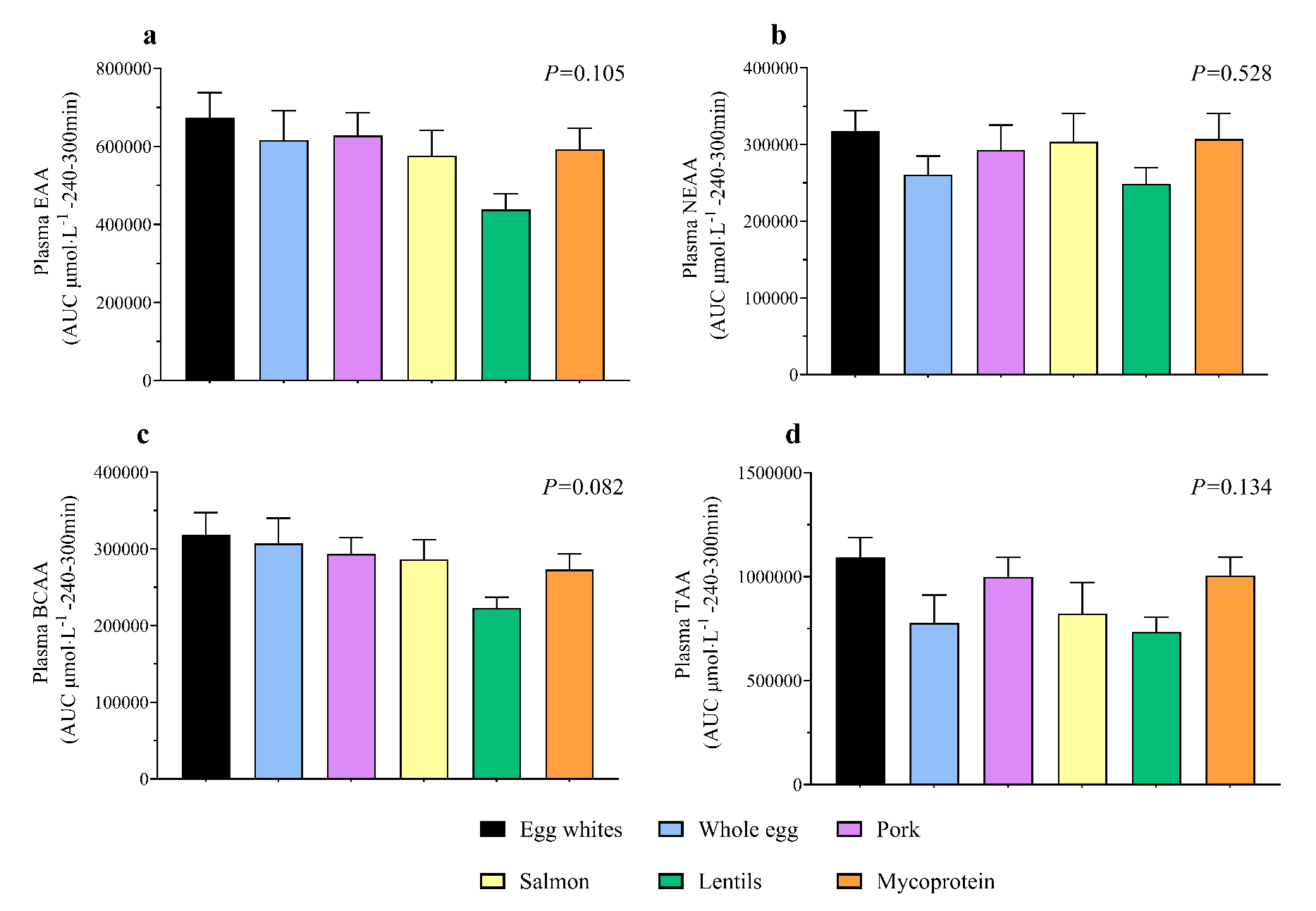
**
