# Supplementary material for: Ingestion of diverse protein-rich whole-foods result in similar post exercise whole body and myofibrillar protein synthesis rates compared with a more isolated protein source in young adults
Source: Am J Clin Nutr. 2026 Feb 3;123(4):101231. doi: 10.1016/j.ajcnut.2026.101231 (PMC13084606; doi:10.1016/j.ajcnut.2026.101231)
Supplement: Multimedia component 4 [file mmc4.docx]

**Ingestion of diverse protein-rich whole-foods result in similar post-exercise whole-body and myofibrillar protein synthesis rates compared with a more isolated protein source in young adults**

Freyja AD Haigh

Online Supplementary Material

**Supplementary Material 3 – Nutritional Analyses Methodology**

Supplied by: Premier Analytical Services | Lincoln Road | High Wycombe | Bucks | HP12 3QS [www.paslabs.co.uk](http://www.paslabs.co.uk)

**Amino acids**

In order to determine the total amino acid content of a given sample, the protein chains must be broken down to its constituent parts. The samples are heated under acidic conditions 115 degrees to hydrolyse the protein chains. For the analysis of cysteine and methionine, the samples are oxidised with performic acid prior to acid hydrolysis. The resulting hydrolysate is diluted, filtered and the pH adjusted.

The extracted amino acids are then derivatised prior to determination by gradient HPLC with fluorescence detection.

This method is UKAS accredited.

**Free amino acids**

Free amino acids are extracted into dilute hydrochloric acid and cleaned up by Carrez reagent. Sample extracts are derivatised with phthalic dicarboxaldehyde (OPA) and / or 3- mercaptoproprionic acid (3-MPA) and analysed by reverse-phase HPLC with fluorescence detection. Each amino acid is quantified by an internal standard method using norvaline and 2-amino adipic acid as internal standards.

**Total Fat (by Nuclear Magnetic Resonance)**

When hydrogen protons are placed in a magnetic field they acquire the ability to resonate when irradiated with radio waves of the correct frequency. When the magnetic field and the radio frequency are correctly matched, the protons absorb and re-emit the radio energy. By detecting the emitted energy and measuring its intensity, the resonating protons in the sample can be measured. Signals from protons in solids have short durations whereas signals from protons in liquids last much longer. As samples are dried prior to analysis to remove moisture and then equilibrated at elevated temperature to melt out any solid fats, the NMR can quantitatively measure the liquid portion and hence the fat content.

**Determination of Aluminium, Calcium, Copper, Iron, Magnesium, Manganese, Potassium, Sodium and Zinc by Inductively Coupled Plasma-Optical Emission Spectrometry (ICP-OES)**

Premier Analytical Services uses the UKAS accredited method C-TM-206 for the Determination of Aluminium, Calcium, Copper, Iron, Magnesium, Manganese, Potassium, Sodium and Zinc by Inductively Coupled Plasma-Optical Emission Spectrometry (ICP-OES) in raw materials and food products. Samples are digested in concentrated nitric acid, which removes organic matter by oxidation. Elemental concentration in the resulting solution is measured using ICP-OES.

**Dietary Fibre**

Premier Analytical Services uses the UKAS accredited method C-TM-129 for the Determination of Dietary Fibre by the AOAC method using MES-TRIS Buffer in raw materials and food products. Starch and protein are removed from the samples enzymatically and the resulting residue, which is insoluble in 78% ethanol, is determined gravimetrically. Corrections are applied to account for residual protein and inorganic components. Modifications are included for the determination of insoluble and soluble dietary fibre.

**Cholesterol**

The method is based upon JAOAC International, Vol. 76, No. 4, 1993, pp902 – 906. Principle of method: The sample is saponified with alcoholic potassium hydroxide. The non-saponifiable fraction is extracted in hexane and then concentrated. Cholesterol is then quantified by gas chromatography.

**Vitamins**

**Water Soluble Vitamins**

**Vitamin B1 (Thiamine)**

Thiamin is extracted by digestion with hydrochloric acid under autoclave conditions, followed by incubation with an α-amylase. Thiamine is separated by ion-pair HPLC and, after post-column reaction with sodium hydroxide/ferricyanide, its concentration is determined as thiochrome using fluorescence detection.

LOQ = 0.05 mg/100g / Rel. Uncertainty* = 12.2%

**Vitamin B2 (Riboflavin)**

Riboflavin is extracted by digestion with hydrochloric acid under autoclave conditions, followed by incubation with an enzyme mixture containing both phosphatase and amylase activity. Riboflavin is separated by ion-pair HPLC and its concentration determined using fluorescence detection.

LOQ = 0.03 mg/100g / Rel. Uncertainty* = 9.5%

**Vitamin B3 (Niacin)**

Total Vitamin B3 (Nicotinamide and Nicotinic acid) are extracted by digestion with hydrochloric acid under autoclave conditions. Nicotinamide and Nicotinic acid are separated by HPLC and their concentration determined by MS-MS using isotopic internal standards.

LOQ = 0.1 mg/100g / Rel. Uncertainty* = 7.7%

**Vitamin B5 (Pantothenic acid)**

Pantothenic acid is extracted by heated digestion in a mildly acidic ammonium acetate buffer and heat-stable α-amylase, followed by dilution with water into the concentration range of the standards. Its concentration is determined by reverse-phase HPLC with MS/MS detection.

LOQ = 0.05 mg/100g / Rel. Uncertainty* = 13.7%

**Vitamin B6**

Vitamin B6 (pyridoxine, pyridoxal and pyridoxamine) is extracted by digestion with hydrochloric acid under autoclave conditions, followed by incubation with an α-amylase. Vitamin B6 forms are separated by ion-pair HPLC and their concentration determined using fluorescence detection.

LOQ = 0.05 mg/100g Rel. Uncertainty* = 4.0%

**Folic Acid (Vitamin B9 – added synthetic form)**

Free folic acid is extracted with ammonium acetate buffer in the presence of mercaptoethanol as antioxidant and its concentration is determined by LCMS/MS.

LOQ = 20 µg/100g / Rel. Uncertainty* = 11.2%

**Vitamin B12 (Cyanocobalamin)**

Total vitamin B12 is determined as cyanocobalamin, the form used for fortification of foods, using reaction with cyanide to convert naturally occurring cobalamins to the cyano form. Cyanocobalamin is determined by LC-MS/MS.

LOQ = 0.1 µg/100g / Rel. Uncertainty* = 14.3%

**Vitamin C**

The vitamin C content is determined as the sum of the ascorbic acid (AA) and dehydroascorbic acid (DHAA) levels. These compounds are extracted into an EDTA/sulphuric acid solution, the AA oxidised to DHAA, the total DHAA derivatised and measured by high performance liquid chromatography with fluorescence detection.

LOQ = 0.1 mg/100g / Rel. Uncertainty* = 17.2% based on spike recovery

**Fat Soluble Vitamins**

**Vitamin A (Retinol)**

Samples are saponified with alcoholic KOH and the retinol is extracted into hexane. After evaporation of the hexane, retinol is re-dissolved in ethanol and its concentration is determined by reverse phase HPLC with UV detection.

LOQ = 10 µg/100g / Rel. Uncertainty* = 11.5%

**Vitamin A (Carotenes) (not UKAS accredited**)

Samples are saponified with alcoholic KOH and carotenes extracted into hexane. The alpha- and ß-carotenes are determined using reverse phase HPLC with visible detection.

LOQ = 10 µg/100g / Rel. Uncertainty* = 8.1% Updated Aug 2024

**Vitamin D**

Vitamin D3 is determined in the absence of vitamin D2 using vitamin D2 as internal standard. Alternatively, vitamin D2 may be determined in the absence of vitamin D3 using vitamin D3 as internal standard. Samples are saponified with alcoholic potassium hydroxide and vitamin D2 (ergocalciferol) and vitamin D3 (cholecalciferol) are extracted into hexane. Their concentrations are determined using HPLC with UV detection.

LOQ = 0.3 µg/100g / Rel. Uncertainty* = 6.9%

**Vitamin E (Tocopherols)**

Vitamin E activity is measured by determining α-, β- and γ- tocopherol content. Samples are saponified with alcoholic KOH and the tocopherols are extracted into hexane. After evaporation of the hexane, tocopherols are redissolved in ethanol and their concentration determined by reverse phase HPLC with fluorescence detection.

LOQ = 0.5 mg/100g / Rel. Uncertainty* = 8.9%

* Relative uncertainty is based on QC test data (unless otherwise stated) and is 2x standard deviation/mean value expressed as percentage.

**Sub-contracted Methods**

Total Folates

Vitamin B9 may be made up of natural folates of plant or animal origin, or added folic acid, which is a synthetic form of the vitamin. Our in-house method for folic acid only measures this added form and is appropriate for fortified foods. Where natural folates are present we sub-contract to a lab that uses LC-MS to determine both natural and added forms of folate.

Biotin (Vitamin B7) The uncertainty of measurement for biotin, which is a microbiological turbidity assay, is 16.3%.

**Available Carbohydrate**

Calculated as 100 - (moisture + ash + protein + fat + dietary fibre)

**Total Carbohydrate**

Calculated as 100 - (moisture + ash + protein + fat)

**Starch**

Calculated by Available Carbohydrate – sugars.
