# Supplementary material for: Ingestion of diverse protein-rich whole-foods result in similar post exercise whole body and myofibrillar protein synthesis rates compared with a more isolated protein source in young adults
Source: Am J Clin Nutr. 2026 Feb 3;123(4):101231. doi: 10.1016/j.ajcnut.2026.101231 (PMC13084606; doi:10.1016/j.ajcnut.2026.101231)
Supplement: Multimedia component 5 [file mmc5.docx]

**Ingestion of diverse protein-rich whole-foods result in similar post-exercise whole-body and myofibrillar protein synthesis rates compared with a more isolated protein source in young adults**

Freyja AD Haigh

Online Supplementary Figure Legends

**Online Supplementary Figure Legends**

**Supplementary Figure 1.** Participants’ Consort flow diagram

**Supplementary Figure 2.** Temporal incremental area under the curve iAUC of serum insulin concentrations over the early (0-120 min) and late (120-300 min) postprandial period in young healthy resistance trained males and females. The vertical dashed line indicating the transition from postabsorptive to postprandial conditions following the ingestion of 0.25g per kg of body mass of egg whites (n=11), whole egg (n=11), pork (n=11), salmon (n=10), lentils (n=11) or mycoprotein (n=11), following a single bout of bilaterial lower body resistance. Data were analyzed using a one-way ANOVA. iAUC, incremental AUC. *, represents a significant difference between food sources. Values are presented as means, with their SEMs represented by vertical bars.

**Supplementary Figure 3.** Time course and incremental area under the curve iAUC of plasma essential amino acid concentrations of leucine (a and b), lysine (c and d), histidine (e and g), threonine (g and h), methionine (i and j), isoleucine (k and l), valine (m and n) during 4 h postabsorptive period (time course graphs only) and a 300 min postprandial period in young healthy resistance trained males and females. iAUC data represent total 300min, 0-120min and 120-300 min postprandial plasma concentrations above postabsorptive values. The vertical dashed line on each graph indicates the transition from postabsorptive to postprandial conditions via the ingestion of 0.25g per kg of body mass of egg whites (n=11), whole egg (n=11), pork (n=11), salmon (n=10), lentils (n=11) or mycoprotein (n=11), following a single bout of bilaterial lower body resistance exercise. Time course data were analyzed with a repeated measures 2-way ANOVA (group x time) with Tukey post hoc tests applied to detect differences at individual time points, found in Supplementary Material Table 6 and 7. Total (0-300 min) iAUC data were analyzed using a one-way ANOVA. Temporal iAUC data (0-120- & 120-300 min) were analyzed using 2-way ANOVA (group x time) with Tukey post hoc tests applied to detect differences at individual time points. *, represents a significant difference between food sources. ɣ, represents a significant difference between time points (0-120 and 120-300 min). Values are presented as means, with their SEMs represented by vertical bars.

**Supplementary Figure 4.** Time course and incremental area under the curve iAUC of plasma non-essential amino acid concentrations of alanine (a and b), glutamic acid (c and d), serine (g and h), proline (i and j) during 240 min postabsorptive period (time course graphs only) and a 300min postprandial period in young healthy resistance trained males and females. iAUC data represent total 300 min, 0-120 min and 120-300 min postprandial plasma concentrations above postabsorptive values. The vertical dashed line on each graph indicates the transition from postabsorptive to postprandial conditions via the ingestion of 0.25g per kg of body mass of egg whites (n=11), whole egg (n=11), pork (n=11), salmon (n=10), lentils (n=11) or mycoprotein (n=11), following a single bout of bilaterial lower body resistance exercise. Time course data were analyzed with a repeated measures 2-way ANOVA (group x time) with Tukey post hoc tests applied to detect differences at individual time points, found in Supplementary Material Table 6 and 7. Total (0-300 min) iAUC data were analyzed using a one-way ANOVA. Temporal iAUC data (0-120- & 120-300 min) were analyzed using 2-way ANOVA (group x time) with Tukey post hoc tests applied to detect differences at individual time points. *, represents a significant difference between food sources. ɣ, represents a significant difference between time points (0-120 and 120-300 min). Values are presented as means, with their SEMs represented by vertical bars.

**Supplementary Figure 5.** Area under the curve (AUC) of serum insulin concentrations in young healthy resistance trained males and females. Data presented includes postabsorptive and postprandial conditions following the ingestion of 0.25g per kg of body mass of egg whites (n=11), whole egg (n=11), pork (n=11), salmon (n=10), lentils (n=11) or mycoprotein (n=11), following a single bout of bilaterial lower body resistance. Data were analyzed using a one-way ANOVA.

**Supplementary Figure 6.** Area under the curve (AUC) of Plasma EAA (a), NEAA (b), BCAA (c) and TAA (d) in young healthy resistance trained males and females. Data presented includes postabsorptive and postprandial conditions following the ingestion of 0.25g per kg of body mass of egg whites (n=11), whole egg (n=11), pork (n=11), salmon (n=10), lentils (n=11) or mycoprotein (n=11), following a single bout of bilaterial lower body resistance. Data were analyzed using a one-way ANOVA. EAA essential amino acids, NEAA non-essential amino acids, BCAA branched chain amino acids, TAA total amino acids.
